# Identifying risk factors for venous thromboembolism in medical inpatients: a systematic review and meta-analysis

**DOI:** 10.1016/j.rpth.2026.106625

**Published:** 2026-04-30

**Authors:** Rachel E. Clapham, Victoria Speed, Catrin Cox, Jignesh P. Patel, Lara N. Roberts

**Affiliations:** 1 King’s Thrombosis Centre, Department of Haematological Medicine, King’s College Hospital NHS Foundation Trust, UK; 2 Faculty of Life Sciences and Medicine, Institute of Pharmaceutical Science, King’s College London, London, UK; 3 Bennett Institute for Applied Data Science, Nuffield Department of Primary Care Health Sciences, University of Oxford, UK; 4 Department of Haematology, University College London Hospitals NHS Foundation Trust, UK

**Keywords:** medical inpatients, prevention, risk factors, thromboprophylaxis, veneous thromboembolism

## Abstract

**Background:**

Hospitalized medical patients are at increased risk of venous thromboembolism (VTE). Current VTE–risk assessment models do not adequately identify which patients would benefit most from prophylaxis, with some low-risk individuals receiving unnecessary anticoagulation and some high-risk patients receiving inadequate protection. A clearer understanding of the risk factors most strongly associated with VTE in this population is needed.

**Objectives:**

A systematic review and meta-analysis were conducted to identify risk factors associated with VTE in acutely ill medical patients.

**Methods:**

The review was registered on the PROSPERO international prospective register of systematic reviews (CRD42024584005). We searched electronic databases from inception to April 2025 for studies that analyzed risk factors associated with VTE in hospitalized medical patients or within 90 days of discharge. Methodological quality was assessed using the Risk of Bias in Nonrandomized Studies—of Interventions tool, and the certainty of evidence for each risk factor was evaluated using the Grading of Recommendations, Assessment, Development, and Evaluations (GRADE) approach. Findings were summarized narratively and by performing a random-effects meta-analysis.

**Results:**

Of 3804 studies identified, 18 met the inclusion criteria. Risk factor associations were reported using regression analyses, nonparametric tests, and descriptive statistics. A history of cancer or current cancer diagnosis; a history of VTE; acute infection; and comorbidities, including diabetes, atrial fibrillation, ischemic stroke, and hypertension, were the most frequently reported risk factors positively associated with VTE. All studies were assessed as having moderate or serious risk of bias. Using the GRADE approach, a history of VTE was the only risk factor graded as having moderate-certainty evidence.

**Conclusion:**

Further work is needed to improve our understanding of which VTE-risk factors are most relevant to medical inpatients.

## Essentials

• Risk factors for VTE in hospitalized medical patients remain uncertain.
• We conducted a systematic review and meta-analysis of studies focused on medical inpatients.
• Cancer, previous VTE, and infection were most consistently associated with VTE.
• Only previous VTE demonstrated moderate-certainty evidence. Further work is required.

## 1 Introduction

Venous thromboembolism (VTE), which includes pulmonary embolism (PE) and deep vein thrombosis (DVT), affects >1 in 12 individuals over the course of their lifetime [1]. Approximately 60% of VTE events are provoked by hospitalization, occurring either during admission or within 90 days postdischarge [2]. Hospitalized medical patients are at particularly high risk, with an estimated 8-fold increase in incidence compared with the general population [3].

Landmark trials of pharmacologic thromboprophylaxis in acutely ill medical patients have demonstrated that it can reduce the risk of hospital-associated VTE by up to 60% [[4], [5], [6]]. However, the applicability of these findings to current medical inpatient cohorts is uncertain, as enrolment was limited to those with specific medical diagnoses (eg, heart failure), and advances in medical care have led to substantial reductions in length of hospital stay. In clinical practice, it is increasingly recognized that some low VTE–risk patients may be unnecessarily receiving pharmacologic thromboprophylaxis, exposing them to potential bleeding complications, while high-risk patients may receive inadequate thromboprophylaxis [7]. This highlights the need to identify which risk factors most strongly predict VTE in medical inpatients.

VTE-risk assessment models (RAMs) can help to decide an individual’s risk of VTE and bleeding, and guide decisions regarding the use of pharmacologic thromboprophylaxis [8]. In the United Kingdom, the National Institute for Health and Care Excellence does not endorse a specific RAM but recommends the use of a published tool to support clinical decision making [9]. Many UK hospitals use the Department of Health VTE RAM, developed for both medical and surgical patients, based on expert consensus [10]. Internationally, the American Society of Hematology similarly does not recommend a single tool but highlights the Padua Prediction Score and the International Medical Prevention Registry on Venous Thromboembolism (IMPROVE) model as commonly used approaches [8]. To date, no RAM has been universally accepted as the optimal tool for VTE prevention in hospitalized medical patients [7]. A systematic review from 2021 of 51 studies comparing RAMs in hospitalized patients found wide variation in study design, implementation of RAMs, outcome definitions, and thromboprophylaxis use, with nearly half of the studies not reporting thromboprophylaxis at all [11]. This highlights limitations in existing models, raising concerns that some relevant risk factors specific to this population may be missing. These limitations are further highlighted by patients classified as high risk who still develop VTE despite thromboprophylaxis.

A previous review has reported on VTE risk in medical patients [12], but its inclusion of broader hospitalized populations limits the applicability of its findings. We therefore conducted a systematic review to identify risk factors associated with VTE in acutely ill medical patients.

## 2 Methods

The systematic review was conducted in accordance with the general principles recommended in the Preferred Reporting Items for Systematic Reviews and Meta-Analyses (PRISMA) statement [13] and was registered on the PROSPERO, an international prospective register of systematic reviews (CRD42024584005).

### 2.1 Data sources and search strategy

We searched for potentially relevant studies through Medline and EMBASE databases and the Cochrane library from inception to April 2025. Searches were supplemented by hand-searching the reference lists of all relevant studies, discussions with expert collaborators, and undertaking systematic keyword searches of the internet via the Google search engine. Search alerts were also set up and monitored to identify and incorporate newly published research.

The search strategy used free text and thesaurus terms and combined synonyms relating to the search question. No date or language restrictions were used on any database. Google Translate was used to assess the eligibility of non-English language articles for inclusion.

### 2.2 Study selection

All titles were initially screened for relevance by 1 reviewer (R.E.C.). Citations clearly unrelated to the topic (eg, not focused on VTE or concerning vitamin K antagonists monitoring or thrombolysis treatment outcomes) were excluded. Title and abstracts were then reviewed independently by 2 reviewers (R.E.C. and V.S.). Full-text articles were subsequently assessed independently (R.E.C. and C.C.) to determine suitability for inclusion, with reasons for exclusion documented for articles excluded at this stage. Discrepancies at any stage were resolved through discussion with a third reviewer (J.P.P.). Covidence, a web-based systematic review platform, was used to facilitate the screening process [14].

Studies were considered eligible for inclusion if they analyzed data on individual risk factors associated with DVT or PE in hospitalized medical patients or associated with hospitalization within 90 days of hospital discharge. Studies that focused on surgical, trauma, or critical care patients; COVID-19–positive patients; pediatric patients (< 18 years old); or nonhospitalized patients were excluded. See Table 1 for the complete inclusion and exclusion criteria.

**Table 1:** Inclusion and exclusion criteria.

Criteria	Inclusion	Exclusion
Population	Hospitalized acutely ill medical patients (>18 y of age) or within 3 mo of discharge	Surgical, trauma, and critical care patients; patients with COVID-19; obstetric patients; pediatric patients; patients discharged from accident and emergency; and nonhospitalized patients
		Studies which only focus on patients with a specific cancer diagnosis
Exposure	VTE-risk factors	—
Outcomes	Symptomatic or asymptomatic deep vein thrombosis or pulmonary embolism	—
Study design	Any study design	—
Time	Any time period	—
Language	Any language	—

### 2.3 Data extraction and risk of bias

A data extraction form was piloted and refined based on feedback from a reviewer (J.P.P.). The final version was used to extract information on study design, methodology, patient demographics, and outcomes. All extracted data were independently verified for accuracy by a second reviewer (J.P.P.), with discrepancies resolved through discussion to achieve consensus. Missing information was recorded as not reported.

The methodological quality of each study was assessed using the Risk of Bias in Nonrandomized Studies—of Interventions (ROBINS-I) tool. This tool provides a structured framework for evaluating risk of bias in nonrandomized studies and is commonly used within systematic reviews [15]. Risk of bias assessments were initially completed by R.E.C. and then independently checked by reviewer J.P.P. Any differences in judgment were discussed and resolved by consensus.

All studies were assessed using the ROBINS-I tool [15], regardless of whether the original study design included randomization to other exposures, to ensure that risk of bias was assessed specifically for the factors relevant to this review. To assess the certainty of evidence for each risk factor, the Grading of Recommendations, Assessment, Development, and Evaluations (GRADE) approach was applied [16]. This method begins by considering the study design: randomized controlled trials (RCTs) are initially rated as high quality, while observational studies as low quality. The certainty rating can then be downgraded based on 5 domains: risk of bias, inconsistency, imprecision, indirectness, and publication bias. Based on these considerations, the overall certainty of evidence for each risk factor is classified as high, moderate, low, or very low [17].

### 2.4 Data synthesis and analysis

Positive and negative effect estimates reported as odds ratios (OR), hazard ratios (HRs), risk ratios (RRs), and adjusted risk ratios (aRRs) were extracted from the included studies. To enable pooling, effect measures that could be converted were treated as approximations of relative risk and transformed into log ORs (log [OR]). When 95% CIs were reported, SEs were calculated directly from the CIs. If only a *P* value was provided, the SE was derived from this. Studies reporting RR or aRR but without sufficient information to convert the estimate to an OR scale were excluded from the meta-analysis. Statistical heterogeneity was assessed using Cochran *Q* test and quantified with the *I*^2^ statistic. All meta-analyses were conducted in R software (version 4.2.2; R Foundation for Statistical Computing) using the meta package (version 8.2.1), and forest plots were generated with the forestplot package (version 3.1.7). Assessment of publication bias (eg, funnel plots or Egger test) was not performed because each pooled analysis included < 10 studies, making these methods unreliable.

For risk factors where meta-analysis was not possible, a descriptive analysis was undertaken using Microsoft Excel 2024. Both positive and negative associations, including nonsignificant results, were extracted. Extracted data included study design, patient characteristics, and risk factors identified in univariable and multivariable analyses.

## 3 Results

**Figure 1:**
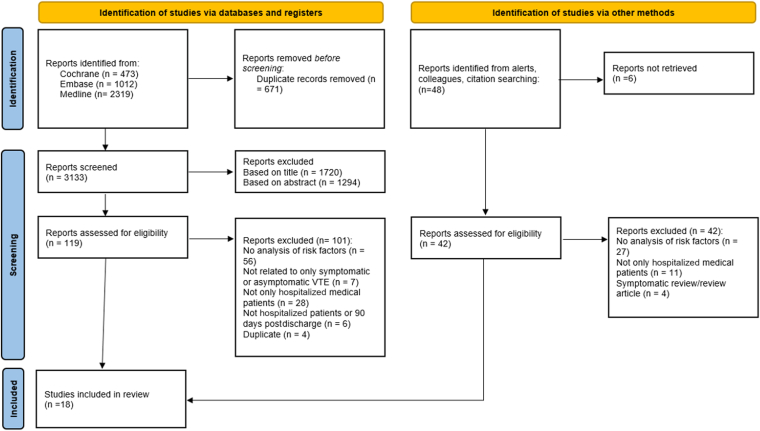
Results of the search strategy using Preferred Reporting Items for Systematic Reviews and Meta-Analyses (PRISMA) 2020 flow diagram. VTE, venous thromboembolism.

A total of 3804 studies were identified through electronic database searching from inception to April 2025 (search strategy available in Supplementary Table 1). After removing duplicates, 3133 titles and abstracts were screened, resulting in 119 articles suitable for full-text review. Of these, 18 studies met the eligibility criteria [[18], [19], [20], [21], [22], [23], [24], [25], [26], [27], [28], [29], [30], [31], [32], [33], [34], [35]] (Table 2). A list of the 101 excluded studies, along with reasons for exclusion, is provided in Supplementary Table 2. Additional studies identified through search alerts, reference screening, and discussions with colleagues (*n* = 48) were also reviewed, but none met the eligibility criteria (Supplementary Table 3). Non-English language articles were translated using Google Translate. The study selection process is presented in the PRISMA flow diagram [36] (Figure 1).

**Table 2:** Summary of included studies.

First author, year, country	Study design, single center/multicenter (No. of centers)	Time frame	Population; total numbers	Males (%)	Age (y), mean (SD)	Inclusion criteria	No. of VTE events	Received prophylaxis, total %	Duration of follow-up (d)	VTE outcome period	Factor collected	Statistical analysis
Alikhan et al. [18], 2004, UK, France, Israel, Sweden, Canada	Prospective randomized RCT, multicenter (9)	1996-1998	Medical patients; 866	49.7	NR	➢ 40 y, projected stay in the hospital ≥ 6 d, and who were not immobilized <3 d, HF (New York Heart Association class III or IV), acute respiratory failure that did not require ventilatory support. Other patients had 1 of 3 medical conditions (acute infectious disease without septic shock, an acute rheumatic disorder, or an active episode of IBD) and at least 1 predefined VTE-risk factor (age > 75 y, cancer, a history of VTE, obesity, varicose veins, hormone therapy, and HF or respiratory failure)	102	Placebo (*n* = 288), enoxaparin 20 mg (*n* = 287), enoxaparin 40 mg (*n* = 291), 67	14	In-hospital and postdischarge	NR	Univariable and multivariable logistic regression
Barba et al. [19], 2012, Spain	Retrospective cohort, multicenter (NR)	2006-2007	COPD; 270,840	29.7	73.92 (12.97)	Diagnosis of COPD	3562	NR	NR	In-hospital	CMBD database	Univariable and multivariable logistic regression
Fan et al. [20], 2011, China	Prospective cohort (41)	2006-2007	Medical patients; 458	65	77 ± 7 (NR)	>60 y, immobilized for 3 d, and hospitalized for HF, respiratory failure, acute ischemic stroke, or acute infectious disease	45 (15 asymptomatic)	None	90	In-hospital and postdischarge	Laboratory tests and standard interview	Univariable logistic regression
Faye et al. [21], 2020, USA	Retrospective cohort, multicenter (2048)	2010-2014	IBD; 872,122	43	NR	Diagnosis of ulcerative colitis or Crohn disease	1160	NR	60	Postdischarge	National readmission database	Univariable and multivariable logistic regression
Kaatz et al. [22], 2012, USA	Retrospective cohort, multicenter (NR)	2005-2009	Medical cancer patients; 267,846	NR	NR	Cancer, hospitalized for >2 d, did not undergo surgery, no surgery <30 d before hospitalization, no prior VTE <90 d of the hospitalization. Postdischarge VTE events <60 d were identified by hospital readmission or an emergency room visit with a principal diagnosis of acute VTE.	1839 HAT, 1972 postdischarge VTE	NR	NR	In-hospital and postdischarge	California hospital discharge data	Univariable and multivariable logistic regression
Khalafallah et al. [23], 2016, Australia	Prospective cohort, single center (1)	2012	Medical patients; 986	50.5	68 (16)	Admitted to medical ward during study period	54	Enoxaparin 40 mg (*n* = 283), UFH 10,000 units daily (*n* = 202), 49	90	In-hospital and postdischarge	Hospital records	Univariable and multivariable logistic regression
Le Gal et al. [24], 2023, USA	Retrospective cohort, multicenter (NR)	2010-2019	Medical patients; 174,592	NR	62 (NR)	Medical patients receiving enoxaparin thromboprophylaxis	5238	Enoxaparin (*n* = 174,592), 100	90	In-hospital and postdischarge	Optum database	Univariable and multivariable logistic regression
Lee et al. [25], 2015, Singapore	Retrospective cohort, single center (1)	2004-2011	Acutely ill medical patients; 199,904	NR	NR	Medical patients	1744	NR	NR	In-hospital	Singapore General Hospital discharge database	Univariable and multivariable logistic regression
Li et al. [26], 2011, China	Prospective cohort, multicenter (40)	2006-2007	Acutely ill elderly medical patients; 607	64.3	77.2 ± 7.6 (NR)	≥ 60 y, >3 d of immobilization, and any of the following: HF, respiratory failure, infective diseases, rheumatic diseases, stroke, and ACS, not CCU patients, able to return to the hospital for a follow-up visit in the third week and give informed consent	59	UFH (*n* = 79), LMWH (*n* = 43), warfarin (*n* = 33), 26	90	In-hospital and postdischarge	Hospital records	Chi-square test for evaluating significant differences for categorical variables; *t*-test for comparing continuous variables
Louzada et al. [27], 2015, Canada	Retrospective cohort, single centre (1)	2011-2013	Medical cancer patients; 875	49.5	64.3 (13.5)	Adult patients: any type of active cancer admitted for ≥ 3 d for treatment of an acute medical reason, who received inpatient dalteparin prophylaxis	78	Dalteparin (*n* = NR)	NR	In-hospital	Hospital records	Univariable and multivariable logistic regression
Mebazaa et al. [28], 2014, 52 countries	Randomized, double-blind, active-comparator–controlled RCT, multicenter (556)	2007-2010	Medical HF patients; 2593	51.5	69.5 (10.6)	≥ 40 y and who were hospitalized for an acute medical illness	NR	Enoxaparin (*n* = NR), rivaroxaban (*n* = NR)	35 ± 4	In-hospital and postdischarge	NR	Univariable and multivariable logistic regression
Merrill et al. [29], 2013, USA	Retrospective case–control, single center (1)	2002-2009 (derivation cohort), 2009-2012 (validation cohort)	Medical patients; 64,334 derivation cohort and 601 controls, 20,946 validation cohort	NR	NR	Admitted to medical services	299 derivation cohort; 120 validation cohort	NR	NR	In-hospital	Hospital records, microbiology laboratory database, vital sign data, laboratory values, confirmatory imaging	Logistic regression
Merrill et al. [30], 2015, USA	Retrospective cohort, single center (1)	2009-2012	Medical patients; 20,327	NR	NR	Medical patients	113	NR	NR	In-hospital	Confirmatory imaging, microbiology results, laboratory and vital sign data, medication records	Logistic regression
Rojnuckarin et al. [31], 2011, Thailand	Retrospective cohort and case–control, single center (1)	2007-2009	Medical patients; 1290 cohort study, case–control—41 VTE cases vs 1263	NR	NR	Medical patients with ≥ 1 risk factor, admitted for ≥ 3 d and had not received anticoagulants	27 cohort study, 41 case–control	NR	42	In-hospital and postdischarge	NR	Multivariable analysis
Sartori et al. [32], 2021, Italy	Prospective cohort, single center (1)	2016-2017	Acutely ill medical patients; 500	40.6	78.1 (13.2)	Symptomatic (with at least 1: leg pain, leg swelling, calf cramps, calf redness, and calf warmth), hospitalized in medical wards for acute illness and developed DVT suspicion after admission. Inclusion criteria were acute HF, acute respiratory disease, infection, acute exacerbation phase of an inflammatory disease, stroke, critical limb ischemia, acute pancreatitis, peptic ulcer disease, hepatic decompensation, and diabetic decompensation	112	Enoxaparin 40 mg (*n* = 236), fondaparinux ≤ 2.5mg (*n* = 41), UFH (*n* = 2), 56	NR	In-hospital	NR	Univariable and multivariable logistic regression, Kaplan–Meier
Spyropoulos et al. [33], 2009, USA	Retrospective cohort, multicenter (>90)	2001-2005	Medically ill patients; 158, 325	52.9	58.5 (12.8)	≥ 40 y and hospitalized for cancer, HF, severe infectious diseases, or lung disease	8895	NR	471.2	In-hospital and postdischarge	PharMetrics patient-centric database	Univariable and multivariable logistic regression, Kaplan–Meier
White et al. [34], 2011, USA	Retrospective cohort, multicenter (NR)	2005-2009	Medical patients; 1,739,089	NR	NR	Most recent medical hospitalization that met specified criteria: absence of any cancer diagnosis, length of hospital stay > 2 d, no acute VTE in the prior 3 mo, and no surgical hospitalization within 1 mo	5416	NR	NR	In-hospital	Linked California hospital discharge records	Logistic regression
Woller et al. [35], 2011, USA	Retrospective cohort, multicenter (22 hospitals, >150 clinics)	2000-2007	Medical patients; 143,975	56	62.78 (NR)	≥ 18 y, admitted to internal medicine or medical subspecial ties	5288	NR	NR	In-hospital	Intermountain Healthcare administrative and electronic medical record systems	Logistic regression, bootstrapping

ACS, acute coronary syndrome; CCU, critical care unit; COPD, chronic obstructive pulmonary disease; DVT, deep vein thrombosis; HAT, hospital-associated thrombosis; HF, heart failure; IBD, inflammatory bowel disease; NR, not recorded; RCT, randomized controlled trial; UFH, unfractionated heparin; VTE, venous thromboembolism.

Table 2 summarizes the study design and patient characteristics of the included studies. The studies were published between 2004 and 2023. Ten were full-text articles [[18], [19], [20], [21],^23^,^26^,^28^,^32^,^33^,^35^] and the remainder were unpublished conference abstracts [22,24,25,27,[29], [30], [31],^34^]. Study designs included 2 RCTs [18,28], 4 prospective observational cohort studies [20,23,26,32], 10 retrospective cohort studies [19,21,22,24,25,27,30,[33], [34], [35]], 1 retrospective case–control study [29], and 1 study with both case–control and cohort components [31]. The studies were conducted across a range of countries: Australia (*n* = 1) [23], Canada (*n* = 2) [18,27], China (*n* = 2) [20,26], France (*n* = 1) [18], Israel (*n* = 1) [18], Italy (*n* = 1) [32], Singapore (*n* = 1) [25], Spain (*n* = 1) [19], Sweden (*n* = 1) [18], Thailand (*n* = 1) [31], United Kingdom (*n* = 1) [18], and United States (*n* = 8) [21,22,24,29,30,[33], [34], [35]]. The proportion of male participants ranged from 30% [19] to 65% [20], and the reported mean age ranged from 58.5 [33] to 78.1 years [32]. The use of thromboprophylaxis varied, with 10 studies not reporting any details [19,21,22,25,[29], [30], [31],[33], [34], [35]]. Across all studies, a total of 36,164 VTE events were reported; however, 1 study did not report the exact number of VTE events [28]. Most studies did not report the duration of follow-up [29,30,32,34,35]; among those that did, follow-up ranged from 14 [18] to 471 days [33]. Data on risk factors were mostly collected from electronic patient records or large databases, including national and hospital-level datasets.

### 3.1 Risk of bias

**Figure 2:**
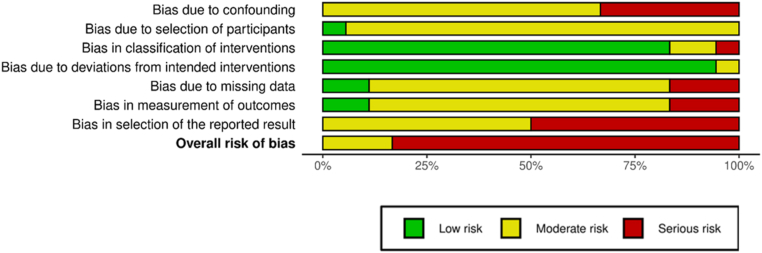
Risk of bias in nonrandomized studies of interventions tool I risk of bias assessment graph.

**Figure 3:**
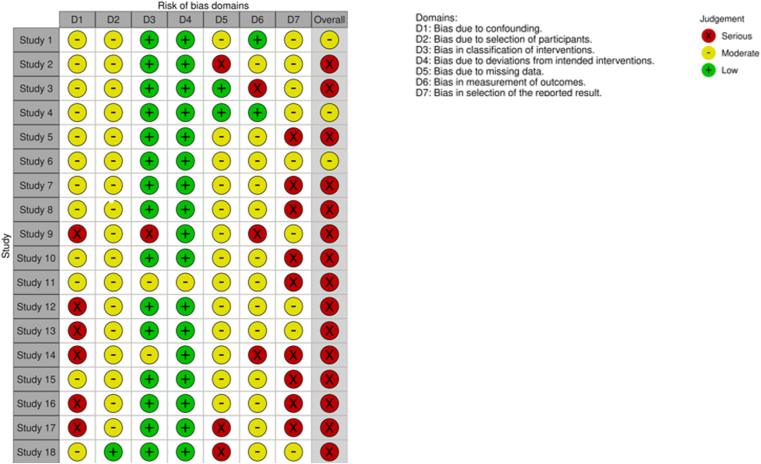
Risk of bias in nonrandomized studies of interventions tool I (ROBINS-I) risk of bias assessment summary.

The overall methodological quality of the 18 included studies was assessed using the ROBINS-I tool [14] (Figures 2 and 3). Fifteen of the 18 studies were found to have an overall serious risk of bias [19,20,22,[24], [25], [26], [27], [28], [29], [30], [31], [32], [33], [34], [35]]. The most frequently observed serious risk of bias was in the selection of the reported result, affecting 9 studies [22,24,25,27,28,[31], [32], [33], [34]]. This was due to the absence of univariable analysis results and missing outcome data. Six studies were also assessed as having a serious risk of bias due to confounding [26,[29], [30], [31],^33^,^34^]. These were observational in nature, and although some attempted to adjust for confounders, missing baseline characteristics limited the effectiveness of these adjustments.

### 3.2 Risk factors positively or negatively associated with development of VTE

The risk factors identified as associated with the development of VTE in multivariable analyses in the reviewed studies are summarized in Table 3. In total, 31 factors were found to be positively associated with PE or DVT (Table 3). A history of cancer or current cancer diagnosis and a history of VTE were the most frequently reported, highlighted in 8 [18,19,22,24,25,31,33,35] and 7 studies [18,21,23,24,[33], [34], [35]], respectively. Fourteen factors were found to be negatively or not associated with PE or DVT (Table 3).

**Table 3:** Risk factors associated with VTE in univariable and multivariable analysis.

Study	Cancer diagnosis/history	VTE history	Acute infection	Comorbidities	Immobility	Length of stay	Respiratory diagnosis	Age	Arterial disease	*Clostridium difficile* test performed or positive result	CVC	D-dimer	Heart failure	Severity of illness	Anticoagulant preadmission	BNP	Compression fracture	CRP	Discharge home with health services or to nursing home	Estrogen	Flexible sigmoidoscopy or colonoscopy on admission	Leg arthritis	Liver disease	Male	Multiple admissions	SLE	Surgery within 30 d	Thrombophilia	Thromboprophylaxis	Varicose vein	Weight
Total No. of studies including risk factor as positive association	8	7	5	5	3	3	3	2	2	2	2	2	2	2	1	1	1	1	1	1	1	1	1	1	1	1	1	1	1	1	1
Total No. of studies including risk factor as negative or no association	3	1	3	2	1	1	5	4	0	0	0	0	4	0	1	0	0	0	0	1	0	0	0	5	0	0	0	0	0	1	3
Alikhan et al. [18], 2004	**✓**	**✓**	**✓**	**–**	**–**	**–**	**○**	**✓**	**–**	**–**	**–**	**–**	**☐**	**–**	**–**	**–**	**–**	**–**	**–**	**☐**	**–**	**–**	**–**	**–**	**–**	**–**	**–**	**–**	**–**	**☐**	**☐**
Barba et al. [19], 2012	**✓**	**–**	**☐**	**✓**	**✓**	**–**	**☐**	**☐**	**✓**	**–**	**–**	**–**	**☐**	**–**	**–**	**–**	**–**	**–**	**–**	**–**	**–**	**–**	**–**	**✓**	**–**	**–**	**–**	**–**	**–**	**–**	**☐**
Fan et al. [20], 2011	**☐**	○	○	**☐**	**–**	**–**	○	**☐**	**–**	**–**	**–**	**✓**	**☐**	**–**	**–**	**–**	**–**	**–**	**–**	**–**	**–**	**–**	**–**	**☐**	**–**	**–**	**–**	**–**	**–**	**–**	**☐**
Faye et al. [21], 2020	**○**	**✓**	**–**	**✓**	**–**	**✓**	**–**	**✓**	**–**	**✓**	**–**	**–**	**–**	**–**	**–**	**–**	**–**	**–**	**✓**	**–**	**✓**	**–**	**–**	**☐**	**–**	**–**	**–**	**–**	**–**	**–**	**–**
Kaatz et al. [22], 2012	**✓**					**✓**								**✓**																	
Khalafallah et al. [23], 2016	**○**	**✓**	**○**	**○**	**☐**	**○**	**✓**	**○**	**–**	**–**	**–**	**–**	**–**	**–**	**☐**	**–**	**–**	**–**	**–**	**–**	**–**	**–**	**–**	**☐**	**–**	**–**	**✓**	**–**	**–**	**–**	**✓**
Le Gal et al. [24], 2023	**✓**	**✓**	**✓**	**✓**			**✓**				**✓**		**✓**		**✓**													**✓**			
Lee et al. [25], 2015	**✓**																						**✓**								
Li et al. [26], 2011			**✓**				**✓**		**✓**				**✓**																		
Louzada et al. [27], 2015	**–**	**–**	**–**	**–**	**–**	**–**	**○**	**–**	**–**	**–**	**–**	**–**	**–**	**–**	**–**	**–**	**–**	**–**	**–**	**–**	**–**	**–**	**–**	**○**	**✓**	**–**	**–**	**–**	**–**	**–**	**–**
Mebazaa et al. [28], 2014												**✓**				**✓**		**✓**													
Merrill et al. [29], 2013										**✓**																					
Merrill et al. [30], 2015			**✓**																												
Rojnuckarin et al. [31], 2011	**✓**		**✓**	**✓**													**✓**			**✓**		**✓**				**✓**				**✓**	
Sartori et al. [32], 2021					**✓**																								**✓**		
Spyropoulos et al. [33], 2009	**✓**	**✓**	**–**	**✓**	**–**	**✓**	**☐**	**☐**	**–**	**–**	**–**	**–**	**☐**	**–**	**–**	**–**	**–**	**–**	**–**	**–**	**–**	**–**	**–**	**☐**	**–**	**–**	**–**	**–**	**–**	**–**	**–**
White et al. [34], 2011		**✓**												**✓**																	
Woller et al. [35], 2011	**✓**	**✓**			**✓**						**✓**																				

BNP, B-type natriuretic peptide; CRP, C-reactive protein; CVC, central venous catheter; SLE, systemic lupus erythematosus; VTE, venous thromboembolism. ✓ indicates a risk factor positively associated with VTE; ○ indicates a risk factor positively associated in the univariable analysis but negatively associated or no association found in the multivariable analysis; **☐** indicates a risk factor negatively associated or no association found in the univariable analysis; – indicates that a risk factors was not considered in the univariable analysis; blank cells indicate it is unknown whether the risk factor was considered in the univariable analysis.

### 3.3 Meta-analysis: cancer

**Figure 4:**
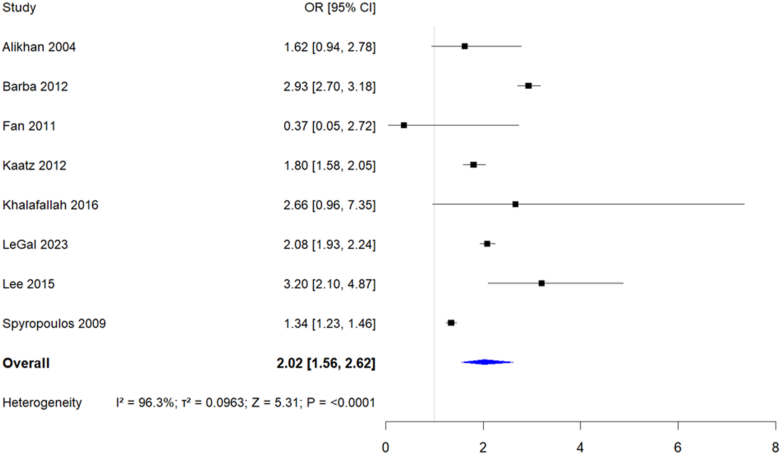
Forest plot showing the association between cancer and venous thromboembolism. OR, odds ratio.

A meta-analysis of 8 of the 11 studies evaluating cancer as a risk factor for VTE [[18], [19], [20],[22], [23], [24], [25],^33^] produced a pooled effect estimate of 2.05 (95% CI, 1.56-2.62), indicating an overall positive association with VTE (Figure 4). Six studies reported a positive association in multivariable analysis [18,19,22,24,25,33], 1 reported a negative association in multivariable analysis [23], and 1 reported a negative association in univariable analysis [20], where a multivariable analysis was not performed. Considerable heterogeneity was observed (OR range, 1.34-3.20; *I*^2^ = 96.3%; *P* < .0001). Of the 3 studies that could not be included in the meta-analysis, 1 reported an increased risk in multivariable analysis (RR, 3.4; *P* = .005) [31]; 1 reported an increased risk in univariable analysis but no association after multivariable adjustment (RR, 1.69; 95% CI, 1.27-2.25) [21]; and 1 reported an increased risk in multivariable analysis but did not provide sufficient statistical detail for inclusion [35].

### 3.4 Meta-analysis: previous VTE

**Figure 5:**
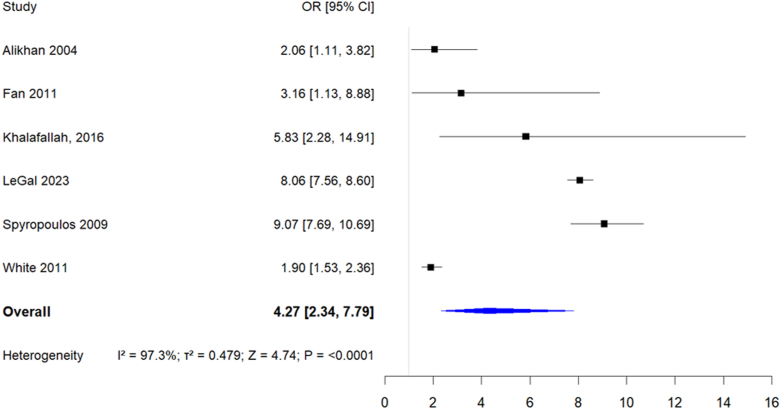
Forest plot showing the association between a history of venous thromboembolism and venous thromboembolism. OR, odds ratio.

Six of the 8 studies investigating previous VTE were included in the meta-analysis [18,20,23,24,33,34], resulting in a pooled effect estimate of 4.27 (95% CI, 2.34-7.79), confirming a strong positive association with VTE (Figure 5). Five studies reported a positive association in multivariable analysis [18,23,24,33,34] and only 1 in univariable analysis [20], as no multivariable analysis was performed. Heterogeneity across these studies was very high (OR range, 2.06-9.06; *I*^2^ = 97.3%) and Cochran *Q* test was statistically significant (*P* < .0001). Among the 2 studies that could not be included in the meta-analysis, 1 reported an elevated risk in multivariable analysis (aRR, 2.41; 95% CI, 1.99-2.90) [21], and the other noted an increased risk in multivariable analysis without providing statistical information [35].

### 3.5 Meta-analysis: acute infection

**Figure 6:**
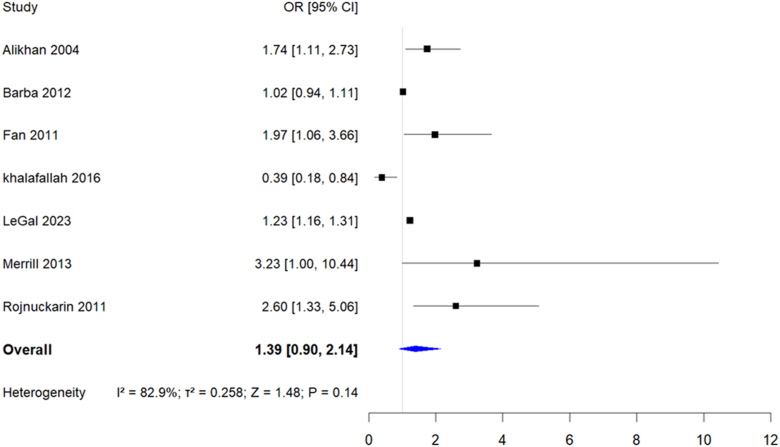
Forest plot showing the association between acute infection and venous thromboembolism. OR, odds ratio.

Seven of the 8 studies [[18], [19], [20],^23^,^24^,^30^,^31^] investigating acute infection were included in the meta-analysis. Most studies reported a positive association in multivariable analysis; however, 1 study was positive only in the univariable analysis and did not do a multivariable analysis [20], 1 reported a negative association in the multivariable analysis [23], and 1 reported no association in the univariable analysis [19]. The pooled effect estimate was 1.39 (95% CI, 0.90-2.14) (Figure 6), indicating no statistically significant overall association. Heterogeneity was high (range of effect estimates: OR, 0.39-3.23; *I*^2^ = 82.9%), although the Cochran *Q* test was not statistically significant (*P* = .14). One additional study could not be included in the meta-analysis due to lack of statistical details but reported an increased risk of VTE in multivariable analysis [26].

### 3.6 Descriptive analysis

The other 28 risk factors could not be analyzed through a meta-analysis. Comorbidities were reported as showing a positive association with the development of VTE in 5 studies and included diabetes, atrial fibrillation, ischemic stroke, and hypertension [19,24], although 1 study did not define which comorbidities were included [21]. One study reported a negative association in univariable analysis [20], and another reported a negative association in multivariable analysis [23]. The remaining risk factors reported a positive association in 3 studies or fewer. However, these findings were inconsistent, with several factors also demonstrating negative or no associations across studies.

### 3.7 Certainty of evidence

The certainty of evidence for each risk factor was assessed using the GRADE approach (Table 4). Only 2 of the included studies were RCT [18,28]; the remainder were observational studies [[19], [20], [21], [22], [23], [24], [25], [26], [27],[29], [30], [31], [32], [33], [34], [35]]. Following the GRADE approach, these observational designs began at a low certainty rating. Risk factors were then assessed for potential downgrading due to risk of bias, inconsistency, indirectness, imprecision, or publication bias [16]. Of the 31 risk factors assessed, 24 were graded as having very low certainty, 6 as low, and 1 as moderate. The only risk factor graded as moderate was a history of VTE, which was evaluated in 1 RCT and multiple observational studies that showed only 1 level of downgrading for risk of bias [18,21,23,24,33,35].

**Table 4:** Certainty of risk factors assessed using GRADE.

Risk factor	Grade
Acute infection	Very low
Age	Very low
Anticoagulant preadmission	Very low
Arterial disease	Very low
BNP	Low
Cancer diagnosis/history	Low
*Clostridium difficile* test performed or positive result	Low
Comorbidities	Very low
Compression fracture	Very low
CRP	Low
CVC	Very low
D-dimer	Very low
Discharge home with health services or to nursing home	Very low
Estrogen	Very low
Flexible sigmoidoscopy or colonoscopy on admission	Very low
Heart failure	Very low
Immobility	Low
Leg arthritis	Very low
Liver disease	Very low
Length of stay	Very low
Male	Very low
Multiple admissions	Very low
Respiratory problem	Low
Severity of illness	Very low
SLE	Very low
Surgery within 30 d	Very low
Thrombophilia	Very low
Thromboprophylaxis	Very low
Varicose veins	Very low
VTE history	Moderate
Weight	Very low

BNP, B-type natriuretic peptide; CRP, C-reactive protein; CVC, central venous catheter; GRADE, Grading of Recommendations, Assessment, Development, and Evaluations; SLE, systemic lupus erythematosus; VTE, venous thromboembolism.

## 4 Discussion

This systematic review of risk factors associated with PE or DVT in hospitalized medical patients found that a history of cancer or a current cancer diagnosis, and a history of VTE, were the most frequently reported (in 8 and 7 studies respectively). Acute infection and comorbidities, including diabetes, atrial fibrillation, ischemic stroke, and hypertension were reported across 5 studies. A history of VTE was the only risk factor, which showed moderate certainty in evidence.

Previous work by Darzi et al. [12], aimed to identify prognostic risk factors in medical patients through a systematic review and meta-analysis [12]. They reported moderate-certainty evidence for several factors, including older age; elevated C-reactive protein, D-dimer, and fibrinogen levels; tachycardia; thrombocytosis; leukocytosis; fever; leg edema; lower Barthel Index score; immobility; paresis; a history of VTE; thrombophilia; malignancy; critical illness; and infections. There were some similarities with our findings, specifically with VTE history, malignancy and infections. When comparing pooled estimates, Darzi et al. [12] reported a stronger association for malignancy (OR, 2.65; 95% CI, 1.79-3.91) and previous VTE (OR, 6.08; 95% CI, 3.71-9.97) than observed in our review (malignancy: OR, 2.05; 95% CI, 1.56-2.62; previous VTE: OR, 4.27; 95% CI, 2.34-7.79). For acute infection, Darzi et al. [12] identified a statistically significant association (OR, 1.48; 95% CI, 1.16-1.89), whereas our pooled estimate was smaller and not statistically significant (OR, 1.39; 95% CI, 0.90-2.14) [12].

The populations included in the review by Darzi et al. [12] differed from ours. They included patients such as those admitted to intensive or critical care units who have distinct risk factors including mechanical ventilation, sedatives, and paralytic drugs [37]. They also included patients with a recent history of surgery of trauma, whose VTE risk is influenced by factors such as the type and complexity of procedure and the type of anesthesia [38]. Additionally, their definition of thromboprophylaxis included agents such as aspirin, which we excluded from consideration [12]. As a result, their findings may include risk factors influenced by these other populations. A limitation of our approach is the exclusion of some recent large RCTs [39,40]; however, this allows us to better understand risk factors unique to medical patients outside of critical or surgical care settings.

While acute infection was generally associated with an increased risk of VTE, 2 studies reported a negative or no association. One study reported a modest reduction in risk (OR, 0.39; 95% CI, 0.18-0.85) [23]; however, the authors suggested that this unexpected finding may have limited clinical relevance, as it could be explained by overlapping diagnostic categories rather than a true protective effect [23]. The other study reported no statistically significant association between acute infection and VTE (OR, 1.02; 95% CI, 0.94-1.11), despite the authors hypothesizing acute infection to be a risk factor.

though the certainty of evidence across the included studies varied, the most frequently reported risks factors in our review are recognized in commonly used VTE RAMs; both the IMPROVE [41] and National Health Service models [10] include a history of VTE and active cancer as key predictors. The CAPRINI score includes present and previous malignancy [42][ as well as a VTE history, whereas the Padua score incorporates active cancer, previous VTE, and acute infection [43]. Their inclusion across these models supports their clinical relevance and their role as important predictors of VTE in medical patients.

Most of the risk factors identified in this systematic review were reported in only 1 or 2 studies and are not included in all currently used VTE-RAMs. However, some may have potential for future incorporation into such models. For example, D-dimer has been evaluated in the IMPROVE–D-dimer tool, developed using data from 7441 hospitalized medically ill patients in the **A**cute Medically Ill Venous Thromboembolism **P**revention with **E**xtended Duration Betri**x**aban (APEX) trial [44]. This model adds 2 points to the original IMPROVE score for a D-dimer level of ≥ 2× the upper limit of normal. Incorporating D-dimer significantly improved risk discrimination and reclassification [44]. Although the APEX trial cohort included intensive care patients, limiting generalizability to strictly medical inpatients, a recent cluster randomized trial of 10,699 reported that implementation of clinical decision support tool incorporating IMPROVE–D-dimer reduced VTE events (2.7% vs 3.3%; OR, 0.80; 95% CI, 0.64-1.00) [45]. The successful integration of D-dimer into an existing model highlights the potential for other emerging risk factors to be incorporated into future VTE RAMs.

These infrequently reported risk factors may still be clinically important for medical patients and warrant consideration. For example, discharge destination, particularly going home with health services or to a nursing home, was reported as a risk factor in only 1 study. Despite its limited appearance in our review, this finding supports existing evidence that nursing home residents are at increased risk of VTE, accounting for 13% of VTE cases in a population-based case–control study conducted in Minnesota, which compared 625 patients with a first diagnosed DVT between 1976 and 1990 to 625 matched controls [46]. These findings also suggest the need for VTE-risk reassessment at discharge to evaluate the potential role of postdischarge prophylaxis.

One of the main limitations of this review was the high level of heterogeneity across the included studies. Meta-analysis was possible for only a small number of risk factors (cancer, previous VTE, and acute infection), and even within these groups, statistical heterogeneity was very high (*I*^2^ > 80%), limiting confidence in the pooled estimates. This heterogeneity likely occurred because most studies were observational in design and varied widely in terms of patient populations, inclusion criteria, and administration of thromboprophylaxis. For the remaining risk factors, there was not enough studies to allow pooling for a meta-analysis. Therefore, the findings were synthesized narratively, which limits the ability to quantify the strength of associations across studies. In addition, variability in study design and reporting meant that uniform definitions for individual risk factors could not be applied. Instead, each risk factor was extracted and reported as described by the original study authors.

In addition, all included studies were assessed as having either moderate or serious risk of bias. These methodological limitations, including selective reporting, missing baseline data, and inadequate adjustment for confounding, reduce the overall strength of evidence for the individual risk factors. Limited transparency around the omission of univariable results in most of the studies also makes it difficult to assess how the variables were selected for multivariable analysis.

Many of the included studies did not report whether patients received pharmacologic thromboprophylaxis. As a result, it was not possible to assess the potential impact of prophylaxis use on VTE incidence or whether patients with specific risk factors were more likely to receive thromboprophylaxis.

Although risk factors may differ between in-hospital and postdischarge VTE, most included studies primarily captured events occurring during hospitalization. Only 1 study reported postdischarge outcomes separately, with the majority analyzing inpatient and postdischarge events together. Therefore, the findings of this review predominantly reflect risk factors identified during the hospital admission period.

Despite the identification of some commonly recognized risk factors, such as active cancer and previous VTE, these findings should be interpreted with caution. The heterogeneity of study populations and the overall inconsistency in risk factor reporting across the studies limits the ability to apply these findings in clinical practice. Although the evidence base is weak, these factors remain the most suitable for further investigation. Our next study will evaluate whether the risk factors identified in this review are predictive of thromboprophylaxis failure. We plan to develop an internally validated predictive model using retrospective data from medical inpatients within our organization, with the aim of potentially improving risk stratification and prevention strategies in hospitalized medical patients.

## 5 Conclusion

A history of cancer or a current cancer diagnosis, a history of VTE, acute infection, and comorbidities were the most frequently reported risk factors for VTE in hospitalized medical patients. However, the overall certainty of evidence was limited, and all included studies were found to be at moderate or serious risk of bias. Further work is needed to improve our understanding of which VTE-risk factors are most relevant to medical inpatients and to support the development of more accurate and clinically meaningful tools for assessing VTE risk.
