# Supplementary material for: Identifying risk factors for venous thromboembolism in medical inpatients: a systematic review and meta-analysis
Source: Res Pract Thromb Haemost. 2026 Apr 30;10(4):106625. doi: 10.1016/j.rpth.2026.106625 (PMC13272504; doi:10.1016/j.rpth.2026.106625)
Supplement: Supplementary Tables S1-S4 [file mmc1.docx]

Supplemental information

Supplemental Table 1. Literature search strategies

| **Database searched:** | **Ovid MEDLINE(R) Epub Ahead of Print, In-Process & Other Non-Indexed Citations, Ovid MEDLINE(R) Daily, Ovid MEDLINE and Versions(R)** |
| --- | --- |
| **Platform or provider used:** | **Ovid SP** |
| **Date of coverage:** | **1946 to October 2024** |
| **Search undertaken:** | **October 2024** |
|  |  |

1. thrombosis.mp. [mp=title, book title, abstract, original title, name of substance word, subject heading word, floating sub-heading word, keyword heading word, organism supplementary concept word, protocol supplementary concept word, rare disease supplementary concept word, unique identifier, synonyms, population supplementary concept word, anatomy supplementary concept word]
2. venous thromb*.mp. [mp=title, book title, abstract, original title, name of substance word, subject

heading word, floating sub-heading word, keyword heading word, organism supplementary concept word, protocol supplementary concept word, rare disease supplementary concept word, unique identifier, synonyms, population supplementary concept word, anatomy supplementary concept word]

1. VTE.ti,ab,kw.
2. embol*.mp. [mp=title, book title, abstract, original title, name of substance word, subject heading word, floating sub-heading word, keyword heading word, organism supplementary concept word, protocol supplementary concept word, rare disease supplementary concept word, unique identifier, synonyms, population supplementary concept word, anatomy supplementary concept word]
3. ((vein* or ven*) adj7 thromb*).ti,ab,kw. *.*
4. thrombus.mp. [mp=title, book title, abstract, original title, name of substance word, subject heading word, floating sub-heading word, keyword heading word, organism supplementary concept word, protocol supplementary concept word, rare disease supplementary concept word, unique identifier, synonyms, population supplementary concept word, anatomy supplementary concept word]
5. "pulmonary embolism*".mp. [mp=title, book title, abstract, original title, name of substance word, subject heading word, floating sub-heading word, keyword heading word, organism supplementary concept word, protocol supplementary concept word, rare disease supplementary concept word, unique identifier, synonyms, population supplementary concept word, anatomy supplementary concept word]
6. deep vein thromb*.mp. [mp=title, book title, abstract, original title, name of substance word, subject heading word, floating sub-heading word, keyword heading word, organism supplementary concept word, protocol supplementary concept word, rare disease supplementary concept word, unique identifier, synonyms, population supplementary concept word, anatomy supplementary concept word]
7. PE.ti,ab,kw.
8. DVT.ti,ab,kw.
9. thromboemboli*.mp. [mp=title, book title, abstract, original title, name of substance word, subject heading word, floating sub-heading word, keyword heading word, organism supplementary concept word, protocol supplementary concept word, rare disease supplementary concept word, unique identifier, synonyms, population supplementary concept word, anatomy supplementary concept word]

12. thromboprophylax*.mp. [mp=title, book title, abstract, original title, name of substance word, subject heading word, floating sub-heading word, keyword heading word, organism supplementary concept word, protocol supplementary concept word, rare disease supplementary concept word, unique identifier, synonyms, population supplementary concept word, anatomy supplementary concept word]

13. prophylax*.mp. [mp=title, book title, abstract, original title, name of substance word, subject heading word, floating sub-heading word, keyword heading word, organism supplementary concept word, protocol supplementary concept word, rare disease supplementary concept word, unique identifier, synonyms, population supplementary concept word, anatomy supplementary concept word]

1. exp Venous Thromboembolism/
2. exp Thromboembolism/
3. exp thrombosis/ or exp venous thrombosis/
4. exp Pulmonary Embolism/

18. 1 or 2 or 3 or 4 or 5 or 6 or 7 or 8 or 9 or 10 or 11 or 12 or 13 or 14 or 15 or 16 or 17

19. risk factor*.mp. [mp=title, book title, abstract, original title, name of substance word, subject heading word, floating sub-heading word, keyword heading word, organism supplementary concept word, protocol supplementary concept word, rare disease supplementary concept word, unique identifier, synonyms, population supplementary concept word, anatomy supplementary concept word]

20. predictive factor*.mp. [mp=title, book title, abstract, original title, name of substance word, subject heading word, floating sub-heading word, keyword heading word, organism supplementary concept word, protocol supplementary concept word, rare disease supplementary concept word, unique identifier, synonyms, population supplementary concept word, anatomy supplementary concept word]

21. prognostic factor*.mp. [mp=title, book title, abstract, original title, name of substance word, subject heading word, floating sub-heading word, keyword heading word, organism supplementary concept word, protocol supplementary concept word, rare disease supplementary concept word, unique identifier, synonyms, population supplementary concept word, anatomy supplementary concept word]

22. patient factor*.mp. [mp=title, book title, abstract, original title, name of substance word, subject heading word, floating sub-heading word, keyword heading word, organism supplementary concept word, protocol supplementary concept word, rare disease supplementary concept word, unique identifier, synonyms, population supplementary concept word, anatomy supplementary concept word]

23. genetic*.mp. [mp=title, book title, abstract, original title, name of substance word, subject heading word, floating sub-heading word, keyword heading word, organism supplementary concept word, protocol supplementary concept word, rare disease supplementary concept word, unique identifier, synonyms, population supplementary concept word, anatomy supplementary concept word]

24. enviromental.mp. [mp=title, book title, abstract, original title, name of substance word, subject heading word, floating sub-heading word, keyword heading word, organism supplementary concept word, protocol supplementary concept word, rare disease supplementary concept word, unique identifier, synonyms, population supplementary concept word, anatomy supplementary concept word]

25. risk assessment*.mp. [mp=title, book title, abstract, original title, name of substance word, subject heading word, floating sub-heading word, keyword heading word, organism supplementary concept word, protocol supplementary concept word, rare disease supplementary concept word, unique identifier, synonyms, population supplementary concept word, anatomy supplementary concept word]

26. demographic factor*.mp. [mp=title, book title, abstract, original title, name of substance word, subject heading word, floating sub-heading word, keyword heading word, organism supplementary concept word, protocol supplementary concept word, rare disease supplementary concept word, unique identifier, synonyms, population supplementary concept word, anatomy supplementary concept word]

27. exp risk assessment/ or exp risk factors/

28. 19 or 20 or 21 or 22 or 23 or 24 or 25 or 26 or 27

29. medical patient*.mp. [mp=title, book title, abstract, original title, name of substance word, subject heading word, floating sub-heading word, keyword heading word, organism supplementary concept word, protocol supplementary concept word, rare disease supplementary concept word, unique identifier, synonyms, population supplementary concept word, anatomy supplementary concept word]

30. medical inpatient*.mp. [mp=title, book title, abstract, original title, name of substance word, subject heading word, floating sub-heading word, keyword heading word, organism supplementary concept word, protocol supplementary concept word, rare disease supplementary concept word, unique identifier, synonyms, population supplementary concept word, anatomy supplementary concept word]

31. hospital medicine.mp. [mp=title, book title, abstract, original title, name of substance word, subject heading word, floating sub-heading word, keyword heading word, organism supplementary concept word, protocol supplementary concept word, rare disease supplementary concept word, unique identifier, synonyms, population supplementary concept word, anatomy supplementary concept word]

32. hospitali?ed medical patient*.mp. [mp=title, book title, abstract, original title, name of substance word, subject heading word, floating sub-heading word, keyword heading word, organism supplementary concept word, protocol supplementary concept word, rare disease supplementary concept word, unique identifier, synonyms, population supplementary concept word, anatomy supplementary concept word]

33. exp Hospitalization/

34. 29 or 30 or 31 or 32 or 33

35. 18 and 28 and 34

36. ((surger* or surgic* or arthro* or endoscop* or op* or "post operative" or "postoper*" or periop* or "peri-op" or preop* or injur* or fracture* or trauma or nephrectomy or gastrectomy or abdominoplasty or hepatectomy or endarterectomy or orthotopic or esophagectomy or ostomy or thromb?ectomy or resternotomy or craniotomy or proctocolectomy or embolectomy or shunt or resection or "spinal fusion" or thromb?elastography or thromboelastometry or "critical care" or "intensive care" or "critically ill" or "ICU" or "covid*" or corona* or SARS or outpatient* or child* or paed* or pediatric or arterial or atrial or arte* or devic* or amputation* or isch?emi* or pregnan* or "post partum" or postpartum or partum or matern* or caesarean or vaginal or hysterectomy or aneurysm* or stenos* or dis?ection or fusion) not medical).ti.

37. 35 not 36

| **Databases searched:** | **EMBASE** |
| --- | --- |
| **Platform or provider used:** | **Ovid SP** |
| **Date of coverage:** | **1974 to October 2024** |
| **Search undertaken:** | **October 2024** |

1. venous thrombo*.ti,kw.
2. thrombosis.mp. [mp=title, abstract, heading word, drug trade name, original title, device manufacturer, drug manufacturer, device trade name, keyword heading word, floating subheading word, candidate term word]
3. VTE.ti,ab,kw.
4. thrombus.mp. [mp=title, abstract, heading word, drug trade name, original title, device manufacturer, drug manufacturer, device trade name, keyword heading word, floating subheading word, candidate term word]
5. ((vein* or ven*) adj7 thromb*).ti,ab,kw.
6. deep vein thromb*.mp. [mp=title, abstract, heading word, drug trade name, original title, device manufacturer, drug manufacturer, device trade name, keyword heading word, floating subheading word, candidate term word]
7. pulmonary embolism*.mp. [mp=title, abstract, heading word, drug trade name, original title, device manufacturer, drug manufacturer, device trade name, keyword heading word, floating subheading word, candidate term word]
8. PE.ti,ab,kw.
9. DVT.ti,ab,kw.
10. thromboemboli*.mp. [mp=title, abstract, heading word, drug trade name, original title, device manufacturer, drug manufacturer, device trade name, keyword heading word, floating subheading word, candidate term word]
11. thromboprophylax*.mp. [mp=title, abstract, heading word, drug trade name, original title, device manufacturer, drug manufacturer, device trade name, keyword heading word, floating subheading word, candidate term word]
12. prophylax*.mp. [mp=title, abstract, heading word, drug trade name, original title, device manufacturer, drug manufacturer, device trade name, keyword heading word, floating subheading word, candidate term word]
13. exp venous thromboembolism/ or exp thromboembolism/
14. exp thrombosis/
15. exp deep vein thrombosis/ or exp lower extremity deep vein thrombosis/
16. exp lung embolism/
17. exp vein thrombosis/
18. exp leg thrombosis/
19. Exp thrombosis prevention/
20. 1 or 2 or 3 or 4 or 5 or 6 or 7 or 8 or 9 or 10 or 11 or 14 or 15 or 16 or 17 or 18 or 19
21. risk factor*.mp. [mp=title, abstract, heading word, drug trade name, original title, device manufacturer, drug manufacturer, device trade name, keyword heading word, floating subheading word, candidate term word]
22. predictive factor*.mp. [mp=title, abstract, heading word, drug trade name, original title, device manufacturer, drug manufacturer, device trade name, keyword heading word, floating subheading word, candidate term word]
23. prognostic factor*.mp. [mp=title, abstract, heading word, drug trade name, original title, device manufacturer, drug manufacturer, device trade name, keyword heading word, floating subheading word, candidate term word]
24. patient factor*.mp. [mp=title, abstract, heading word, drug trade name, original title, device manufacturer, drug manufacturer, device trade name, keyword heading word, floating subheading word, candidate term word]
25. risk assessment*.mp. [mp=title, abstract, heading word, drug trade name, original title, device manufacturer, drug manufacturer, device trade name, keyword heading word, floating subheading word, candidate term word]
26. demographic factor*.mp. [mp=title, abstract, heading word, drug trade name, original title, device manufacturer, drug manufacturer, device trade name, keyword heading word, floating subheading word, candidate term word]
27. exp risk factor/
28. exp risk assessment/
29. 21 or 22 or 23 or 24 or 25 or 26 or 27 or 28
30. medical patient*.mp. [mp=title, abstract, heading word, drug trade name, original title, device manufacturer, drug manufacturer, device trade name, keyword heading word, floating subheading word, candidate term word]
31. hospitali?ed medical patient*.mp. [mp=title, abstract, heading word, drug trade name, original title, device manufacturer, drug manufacturer, device trade name, keyword heading word, floating subheading word, candidate term word]
32. medical inpatient*.mp. [mp=title, abstract, heading word, drug trade name, original title, device manufacturer, drug manufacturer, device trade name, keyword heading word, floating subheading word, candidate term word]
33. exp acutely ill patient/
34. 30 or 31 or 32 or 33
35. 20 and 29 and 34
36. 36. ((surger* or surgic* or arthro* or endoscop* or op* or "post operative" or "postoper*" or periop* or "peri-op" or preop* or injur* or fracture* or trauma or nephrectomy or gastrectomy or abdominoplasty or hepatectomy or endarterectomy or orthotopic or esophagectomy or ostomy or thromb?ectomy or resternotomy or craniotomy or proctocolectomy or embolectomy or shunt or resection or "spinal fusion" or thromb?elastography or thromboelastometry or "critical care" or "intensive care" or "critically ill" or "ICU" or "covid*" or corona* or SARS or outpatient* or child* or paed* or pediatric or arterial or atrial or arte* or devic* or amputation* or isch?emi* or pregnan* or "post partum" or postpartum or partum or matern* or caesarean or vaginal or hysterectomy or aneurysm* or stenos* or dis?ection or fusion) not medical).ti.
37. 35 not 36

| **Databases searched:** | **Cochrane Database of Systematic Reviews, Cochrane Central Register of Controlled Trials, Database of Abstracts of Review of Effects, Health Technology Assessment Database and NHS Economic Evaluation Database** |
| --- | --- |
| **Platform or provider used:** | **Wiley Online** |
| **Date of coverage:** | **1898 to October 2024** |
| **Search undertaken:** | **October 2024** |
|  |  |

1. MeSH descriptor: [Thrombosis] explode all trees
2. MeSH descriptor: [Venous Thrombosis] explode all trees

MeSH descriptor: [Venous Thromboembolism] explode all trees

1. MeSH descriptor: [Pulmonary Embolism] explode all trees
2. (thrombus* or thrombotic* or thrombolic* or thromboemboli* or thrombos* or embol*):ti,ab,kw
3. (vein* or ven*):ti,ab,kw
4. (PE):ti,ab,kw
5. (DVT):ti,ab,kw
6. (VTE):ti,ab,kw
7. thromboprophylax*
8. prophylax*
9. #1 or #2 or #3 or #4 or #5 or #6 or #7 or #8 or #9 or #10 or #11
10. MeSH descriptor: [Risk Factors] explode all trees
11. MeSH descriptor: [Risk Assessment] explode all trees
12. MeSH descriptor: [Demography] explode all trees
13. (risk assess*):ti,ab,kw
14. (predictive factor*):ti,ab,kw
15. (prognostic factor*)ti,ab,kw
16. (demographic factor*):ti,ab,kw
17. (patient factor*):ti,ab,kw
18. #13 or #14 #15 or #16 or #17 or #18 or #19 or #20
19. (medical NEXT patient*):ti,ab,kw
20. (hospitali?ed medical patient*):ti,ab,kw
21. (medical inpatient*):ti,ab,kw
22. #22 or #23 or #24
23. #12 and #21 and #25
24. ((surger* or surgic* or arthro* or endoscop* or op* post NEXT operative or postoper* or periop* or peri-op or preop* or injur* or fracture* or trauma or nephrectomy or gastrectomy or abdominoplasty or hepatectomy or endarterectomy or orthotopic or esophagectomy or ostomy or thromb?ectomy or resternotomy or craniotomy or proctocolectomy or embolectomy or shunt or resection or "spinal fusion" or thromb?elastography or thromboelastometry or critical NEXT care or intensive NEXT care or critically NEXT ill or ICU or covid* or corona* or SARS or outpatient* or child* or paed* or pediatric or neonat* or infant* or labour or arterial or atrial or arte* or devic* or amputation* or isch?emi* or pregnan* or post NEXT partum or postpartum or partum or matern* or caesarean or vaginal or hysterectomy or aneurysm* or pseudoaneurysm* or stenos* or dis?ection or fusion or leg NEXT ulcer*) NOT medical):ti
25. #26 NOT #27

Supplemental Table 2. Excluded database studies with rationale

|  | **Author, year** | **Reason for exclusion** |
| --- | --- | --- |
| 1 | Abimana et al., 2022 | No analysis of risk factors associated with VTE |
| 2 | Ageno et al., 2012a | No analysis of risk factors associated with VTE |
| 3 | Ageno et al., 2012b | No analysis of risk factors associated with VTE |
| 4 | Alikhan et al., 2003 | No analysis of risk factors associated with VTE |
| 5 | Amin et al., 2017 | No analysis of risk factors associated with VTE |
| 6 | Amin et al., 2018a | No analysis of risk factors associated with VTE |
| 7 | Amin et al., 2018b | No analysis of risk factors associated with VTE |
| 8 | Amin et al., 2024 | No analysis of risk factors associated with VTE |
| 9 | Ananthakrishnan et al., 2014 | Outcomes: Not related to only symptomatic or asymptomatic VTE |
| 10 | Andrade et al., 2009 | Population: Not only hospitalised medical patients |
| 11 | Angelini et al., 2015 | No analysis of risk factors associated with VTE |
| 12 | Asmamaw et al., 2022 | No analysis of risk factors associated with VTE |
| 13 | Barbar et al., 2010 | Duplicate |
| 14 | Barclay et al., 2013 | No analysis of risk factors associated with VTE |
| 15 | Bergmann et al., 2011 | No analysis of risk factors associated with VTE |
| 16 | Blondon et al., 2017 | No analysis of risk factors associated with VTE |
| 17 | Blondon et al., 2019 | No analysis of risk factors associated with VTE |
| 18 | Blondon et al., 2020 | No analysis of risk factors associated with VTE |
| 19 | Budd et al., 2021 | No analysis of risk factors associated with VTE |
| 20 | Campanini et al., 2010 | No analysis of risk factors associated with VTE |
| 21 | Campbell et al., 2009 | No analysis of risk factors associated with VTE |
| 22 | Carrier et al., 2023 | Population: Not only hospitalised medical patients |
| 23 | Chen et al., 2015 | Outcomes: Not related to only symptomatic or asymptomatic VTE |
| 24 | Chen et al., 2017 | No analysis of risk factors associated with VTE |
| 25 | Chen et al., 2023 | Population: Not only hospitalised medical patients |
| 26 | Chen et al., 2024 | No analysis of risk factors associated with VTE |
| 27 | Chi et al., 2017 | Population: Not only hospitalised medical patients |
| 28 | Chi et al., 2018a | Population: Not only hospitalised medical patients |
| 29 | Chi et al., 2018b | Population: Not only hospitalised medical patients |
| 30 | Chi et al., 2018c | Population: Not only hospitalised medical patients |
| 31 | Chi et al., 2018d | Population: Not only hospitalised medical patients |
| 32 | Chi et al., 2019 | Population: Not only hospitalised medical patients |
| 33 | Chi et al., 2023 | No analysis of risk factors associated with VTE |
| 34 | Chulkov et al., 2022 | No analysis of risk factors associated with VTE |
| 35 | Cohen et al., 2007 | No analysis of risk factors associated with VTE |
| 36 | Cohen et al., 2010 | No analysis of risk factors associated with VTE |
| 37 | Cohen et al., 2011a | No analysis of risk factors associated with VTE |
| 38 | Cohen et al., 2011b | Population: Not only hospitalised medical patients |
| 39 | Cohen et al., 2011c | No analysis of risk factors associated with VTE |
| 40 | Cohen et al., 2014 | No analysis of risk factors associated with VTE |
| 41 | Cohoon et al., 2018 | No analysis of risk factors associated with VTE |
| 42 | Drozdinsky et al., 2024 | Population: Not only hospitalised medical patients |
| 43 | Edelsberg et al., 2006 | Population: Not only hospitalised medical patients |
| 44 | Ferreira et al., 2021 | Population: Not only hospitalised medical patients |
| 45 | Gal et al., 2024 | Population: Not only hospitalised medical patients |
| 46 | Gerotziafas et al., 2010 | Population: Not only hospitalised medical patients |
| 47 | Gibson et al., 2016 | Population: Not only hospitalised medical patients |
| 48 | Grant et al., 2013 | No analysis of risk factors associated with VTE |
| 49 | Grant et al., 2016 | No analysis of risk factors associated with VTE |
| 50 | Hotoleanu and Andercou, 2014 | No analysis of risk factors associated with VTE |
| 51 | Houghton et al., 2013 | No analysis of risk factors associated with VTE |
| 52 | Hull et al., 2010 | Outcomes: Not related to only symptomatic or asymptomatic VTE |
| 53 | Kalayci et al., 2022 | No analysis of risk factors associated with VTE |
| 54 | Kato et al., 2012 | No analysis of risk factors associated with VTE |
| 55 | Khorana et al., 2007 | Population: Not only hospitalised medical patients |
| 56 | Kolomansky et al., 2006 | Setting: Not hospitalised patients or 3 months post-discharge |
| 57 | Louzada et al., 2014 | No analysis of risk factors associated with VTE |
| 58 | Mahan et al., 2013 | No analysis of risk factors associated with VTE |
| 59 | Miller et al., 2012 | No analysis of risk factors associated with VTE |
| 60 | Monti et al., 2019a | Population: Not only hospitalised medical patients |
| 61 | Monti et al., 2019b | Duplicate |
| 62 | Mottier et al., 2023 | Outcomes: Not related to only symptomatic or asymptomatic VTE |
| 63 | Nafee et al., 2020 | No analysis of risk factors associated with VTE |
| 64 | Neuman et al., 2017 | Outcomes: Not related to only symptomatic or asymptomatic VTE |
| 65 | Novelli et al., 2012 | Setting: Not hospitalised patients or 3 months post-discharge |
| 66 | Perez et al., 2023 | Population: Not only hospitalised medical patients |
| 67 | Piazza et al., 2011 | Population: Not only hospitalised medical patients |
| 68 | Prandoni et al., 2010 | No analysis of risk factors associated with VTE |
| 69 | Rodrigues Lima Ferreira et al., 2019 | No analysis of risk factors associated with VTE |
| 70 | Rojnuckarin et al., 2011 | No analysis of risk factors associated with VTE |
| 71 | Rosenberg et al., 2014 | No analysis of risk factors associated with VTE |
| 72 | Rothberg et al., 2018 | No analysis of risk factors associated with VTE |
| 73 | Rothberg et al., 2022 | Population: Not only hospitalised medical patients |
| 74 | Ryan et al., 2021 | No analysis of risk factors associated with VTE |
| 75 | Sejrup et al., 2019 | Population: Not only hospitalised medical patients |
| 76 | Serrano et al., 2018 | No analysis of risk factors associated with VTE |
| 77 | Shah et al., 2018 | No analysis of risk factors associated with VTE |
| 78 | Silvestri et al., 2013 | No analysis of risk factors associated with VTE |
| 79 | Smilowitz et al., 2019 | Setting: Not hospitalised patients or 3 months post-discharge |
| 80 | Smilowitz et al., 2021 | Outcomes: Not related to only symptomatic or asymptomatic VTE |
| 81 | Sparks et al., 2022 | No analysis of risk factors associated with VTE |
| 82 | Spyropoulos, 2009 | Duplicate |
| 83 | Spyropoulos et al., 2011 | Population: Not only hospitalised medical patients |
| 83 | Spyropoulos et al., 2020 | No analysis of risk factors associated with VTE |
| 84 | Sun et al., 2018 | No analysis of risk factors associated with VTE |
| 85 | Thomas et al., 2023 | No analysis of risk factors associated with VTE |
| 86 | Tsai et al., 2015 | Population: Not only hospitalised medical patients |
| 87 | Vardi et al., 2013 | Outcomes: Not related to only symptomatic or asymptomatic VTE |
| 88 | Vincentelli et al., 2018 | Setting: Not hospitalised patients or 3 months post-discharge |
| 89 | Wang et al., 2014 | Population: Not only medical patients |
| 90 | Wilkinson et al., 2024 | No analysis of risk factors associated with VTE |
| 91 | Woller et al., 2010 | No analysis of risk factors associated with VTE |
| 92 | Woller et al., 2020 | No analysis of risk factors associated with VTE |
| 93 | Wurtz et al., 2020 | Setting: Not hospitalised patients or 3 months post-discharge |
| 94 | Yang et al., 2023 | No analysis of risk factors associated with VTE |
| 95 | Zakai et al., 2004 | No analysis of risk factors associated with VTE |
| 96 | Zakai et al., 2011a | Duplicate |
| 97 | Zakai et al., 2011b | Population: Not only medical patients |
| 98 | Zakai et al., 2012 | Population: Not only medical patients |
| 99 | Zakai et al., 2013 | Setting: Not hospitalised patients or 3 months post-discharge |
| 100 | Zakai et al., 2021 | No analysis of risk factors associated with VTE |
| 101 | Zakai et al., 2024 | Population: Not only medical patients |

1. Abimana, E., Ntabanganyimana, E., Ndahimana, R., Sebatunzi, O.R., Masaisa, F., 2022. Assessment of venous thromboembolism risk and use of anticoagulant thromboprophylaxis in CHUK, Rwanda: a cross-sectional study. medRxiv. https://doi.org/10.1101/2022.12.26.22283948
2. Ageno, W., Riva, N., Noris, P., Di Nisio, M., La Regina, M., Arioli, D., Ria, L., Monzani, V., Cuppini, S., Lupia, E., Giorgi Pierfranceschi, M., Dentali, F., 2012a. Safety and efficacy of low-dose fondaparinux (1.5 mg) for the prevention of venous thromboembolism in acutely ill medical patients with renal impairment: the FONDAIR study. Journal of Thrombosis & Haemostasis 10, 2291–2297.
3. Ageno, W., Riva, N., Noris, P., Di Nisio, M., La Regina, M., Arioli, D., Ria, L., Monzani, V., Cuppini, S., Lupia, E., Giorgi Pierfranceschi, M., Pini, M., Agnelli, G., De Gaudenzi, E., Re, R., Manfredini, R., Vidili, G., Parente, F., Chesi, G., Loffredo, L., Moreo, G., Dentali, F., Squizzato, A., Imberti, D., Silingardi, M., Pattacini, C., Becattini, C., 2012b. Low-dose fondaparinux for the prevention of venous thromboembolism in acutely ill medical patients with renal impairment: The fondair study. Blood Transfusion 10, s41–s42.
4. Alikhan, R., Cohen, A.T., Combe, S., Samama, M.M., Desjardins, L., Eldor, A., Janbon, C., Leizorovicz, A., Olsson, C.-G., Turpie, A.G.G., 2003. Prevention of venous thromboembolism in medical patients with enoxaparin: a subgroup analysis of the MEDENOX study. Blood coagulation & fibrinolysis : an international journal in haemostasis and thrombosis 14, 341–6. https://doi.org/10.1097/00001721-200306000-00004
5. Amin, A., Neuman, W.R., Lingohr-Smith, M., Menges, B., Lin, J., 2017. Venous thromboembolism prophylaxis and risk in the inpatient and outpatient continuum of care among acutely ill medical patients in the US. Pharmacotherapy 37, e142–e143. https://doi.org/10.1002/phar.2052
6. Amin, A., Neuman, R., Lingohr-Smith, M., Menges, B., Lin, J., 2018a. Status of venous thromboembolism (VTE) prophylaxis among patients hospitalized for acute rheumatic diseases in the US. Journal of General Internal Medicine 33, 347.
7. Amin, A., Neuman, W.R., Lingohr-Smith, M., Menges, B., Lin, J., 2018b. Risk for venous thromboembolism in the continuum of care among patients hospitalized for acute medical illnesses in the us. American Journal of Hematology 93, E4. https://doi.org/10.1002/ajh.25268
8. Amin, A.N., Kartashov, A., Ngai, W., Steele, K., Rosenthal, N., 2024. Effectiveness, Safety, and Costs of Thromboprophylaxis with Enoxaparin or Unfractionated Heparin Among Medical Inpatients With Chronic Obstructive Pulmonary Disease or Heart Failure. Journal of Health Economics & Outcomes Research 11, 44–56.
9. Ananthakrishnan, A.N., Cagan, A., Gainer, V.S., Cheng, S.-C., Cai, T., Scoville, E., Konijeti, G.G., Szolovits, P., Shaw, S.Y., Churchill, S., Karlson, E.W., Murphy, S.N., Kohane, I., Liao, K.P., 2014. Thromboprophylaxis is associated with reduced post-hospitalization venous thromboembolic events in patients with inflammatory bowel diseases. Clinical gastroenterology and hepatology : the official clinical practice journal of the American Gastroenterological Association 12, 1905–10. https://doi.org/10.1016/j.cgh.2014.02.034
10. Andrade, E. de O., Binda, F.A., Silva, A.M.M. da, Costa, T.D.A. da, Fernandes, Marcelio Costa, Fernandes, Marcio Costa, 2009. Risk factors and prophylaxis for venous thromboembolism in hospitals in the city of Manaus, Brazil. Jornal brasileiro de pneumologia : publicacao oficial da Sociedade Brasileira de Pneumologia e Tisilogia 35, 114–21. https://doi.org/10.1590/s1806-37132009000200003
11. Angelini, D.E., Greene, T., Wietzke, J.N., Flanders, S.A., Sood, S.L., 2015. Defining the risk: Benefit ratio of venous thromboembolism (VTE) prophylaxis in hospitalized cancer patients. Blood 126, 627.
12. Asmamaw, M., Hungnaw, W., Motbainor, A., Kedir, H.M., Tadesse, T.A., 2022. Incidence of thromboembolism and thromboprophylaxis in medical patients admitted to specialized hospital in Ethiopia using Padua prediction score. SAGE Open Medicine 1
13. Barbar, S., Noventa, F., Rossetto, V., Ferrari, A., Brandolin, B., Perlati, M., De Bon, E., Tormene, D., Pagnan, A., Prandoni, P., 2010. A risk assessment model for the identification of hospitalized medical patients at risk for venous thromboembolism: the Padua Prediction Score. Journal of Thrombosis & Haemostasis 8, 2450–2457.
14. Barclay, S.M., Jeffres, M.N., Nguyen, K., Nguyen, T., 2013. Evaluation of pharmacologic prophylaxis for venous thromboembolism in patients with chronic liver disease. Pharmacotherapy 33, 375–82. https://doi.org/10.1002/phar.1218
15. Bergmann, J.F., Lloret-Linares, C., Rami, A., Cohen, A.T., Garay, R.P., Kakkar, A.K., Goldhaber, S.Z., Deslandes, B., Tapson, V.F., Anderson, F.A., 2011. Venous thromboembolism risk and prophylaxis in the acute hospital care setting (ENDORSE study): Results obtained in France. Presse Medicale 40, e528–e537. https://doi.org/10.1016/j.lpm.2011.06.023
16. Blondon, M., Spirk, D., Kucher, N., Aujesky, D., Hayoz, D., Beer, J., Husmann, M., Frauchiger, B., Korte, W., Wuillemin, W., Bounameaux, H., Righini, M., Nendaz, M., 2017. External validation and comparison of the improve risk assessment model with the Geneva risk assessment model in the estimate cohort. Research and Practice in Thrombosis and Haemostasis 1, 180. https://doi.org/10.1002/rth2.12012
17. Blondon, M., Limacher, A., Righini, M., Aujesky, D., Mean, M., 2019. Adequacy of hospital thromboprophylaxis and risk assessment models in the SWITCO65+ cohort. Research and Practice in Thrombosis and Haemostasis 3, 760. https://doi.org/10.1002/rth2.12229
18. Blondon, M., Righini, M., Nendaz, M., Glauser, F., Robert-Ebadi, H., Prandoni, P., Barbar, S., 2020. External validation of the simplified Geneva risk assessment model for hospital-associated venous thromboembolism in the Padua cohort. Journal of Thrombosis and Haemostasis 18, 676–680. https://doi.org/10.1111/jth.14688
19. Budd, A.C., Rhodes, M., Forster, A.J., Noghani, P., Carrier, M., Wells, P.S., 2021. Prescribing patterns and outcomes of venous thromboembolism prophylaxis in hospitalized medical and cancer patients: Observations from the Ottawa Hospital. Thrombosis Research 197, 144–152. https://doi.org/10.1016/j.thromres.2020.11.013
20. Campanini, M., Gussoni, G., Silingardi, M., Scannapieco, G., Buniolo, C., Valerio, A., Ageno, W., Lori, I., Mazzone, A., 2010. Risk factors for venous thromboembolism and prophylaxis in medical inpatients: Data from the FADOI “GEMINI” study. Italian Journal of Medicine 4, 23–31. https://doi.org/10.1016/j.itjm.2010.01.002
21. Campbell, P., Arbuthnot, C., Reed, M., Passlow, C., Vagg, M., Orford, N., Tomlinson, S., 2009. Prevent - An electronic VTE risk assessment tool and decision support in acute inpatients. Journal of thrombosis and haemostasis : JTH 7, 270‐271. https://doi.org/10.1111/j.1538-7836.2009.03473-1.x
22. Carrier, M., Arcelus, J., Stroh, C., Khan, I., Djoudi, Y., Ponomareva, E., Ageno, W., 2023. Venous Thromboembolism and Major Bleeding Risk in Hospitalized Obese Patients Receiving Thromboprophylaxis with Enoxaparin. Research and Practice in Thrombosis and Haemostasis 7, 101857. https://doi.org/10.1016/j.rpth.2023.101857
23. Chen, X., Huang, J., Liu, J., Chang, J., Pan, L., Wang, Y., Gao, Y., Yang, Y., 2023. Derivation and External Validation of a Risk Assessment Model of Venous Thromboembolism in Hospitalized Chinese Patients. Clinical and applied thrombosis/hemostasis : official journal of the International Academy of Clinical and Applied Thrombosis/Hemostasis 29, 10760296221151164. https://doi.org/10.1177/10760296221151164
24. Chen, X., Shi, H., Chang, J., Guo, W., Yang, Y., Wang, Y., Pan, L., 2024. External Validation of the Risk Assessment Model of Venous Thromboembolism in Multicenter Internal Medicine Inpatients. Clinical & Applied Thrombosis/Hemostasis 1.
25. Chen, Y.-G., Lin, T.-Y., Huang, W.-Y., Lin, C.-L., Dai, M.-S., Kao, C.-H., 2015. Association between pneumococcal pneumonia and venous thromboembolism in hospitalized patients: A nationwide population-based study. Respirology (Carlton, Vic.) 20, 799–804. https://doi.org/10.1111/resp.12501
26. Chen, Y., Zhou, H.X., Hu, Y.H., Cong, T.X., Tang, Y.J., Wang, L., Wang, M.Y., Yi, Q., Liang, Z.A., 2017. [Risk factors of pulmonary embolism in senile and non-senile inpatients and the predictive value of Caprini risk assessment model in these two populations]. Zhonghua yi xue za zhi 97, 755–760. https://doi.org/10.3760/cma.j.issn.0376-2491.2017.10.008
27. Chi, G., Goldhaber, S.Z., Hull, R.D., Hernandez, A.F., Kerneis, M., Al Khalfan, F., Cohen, A.T., Harrington, R.A., Michael Gibson, C., 2017. Thrombus Burden of Deep Vein Thrombosis and Its Association with Thromboprophylaxis and D-Dimer Measurement: Insights from the APEX Trial. Thrombosis and Haemostasis 117, 2389–2395. https://doi.org/10.1160/TH17-08-0538
28. Chi, G., Gibson, C.M., Hernandez, A.F., Hull, R.D., Kalayci, A., Kerneis, M., Alkhalfan, F., Nafee, T., Cohen, A.T., Harrington, R.A., Goldhaber, S.Z., 2018a. Association of low hemoglobin with venous thromboembolism in acutely ill hospitalized medical patients: Findings from the APEX trial. European Heart Journal 39, 318. https://doi.org/10.1093/eurheartj/ehy565.P1623
29. Chi, Gerald, Gibson, C.M., Hernandez, A.F., Hull, R.D., Kazmi, S.H.A., Younes, A., Walia, S.S., Pitliya, A., Singh, A., Kahe, F., Kalayci, A., Nafee, T., Kerneis, M., AlKhalfan, F., Cohen, A.T., Harrington, R.A., Goldhaber, S.Z., 2018b. Association of Anemia with Venous Thromboembolism in Acutely Ill Hospitalized Patients: An APEX Trial Substudy. American Journal of Medicine 131.
30. Chi, G., Gibson, M., Hernandez, A.F., Hull, R.D., Cohen, A.T., Harrington, R.A., Liu, Y., Walia, S., Sharfaei, S., Pitliya, A., Kazmi, H.A., Datta, S., Kahe, F., Ghaffarpasand, E., Jafarizde, M., Kalayci, A., Nafee, T., Kerneis, M., AlKhalfan, F., Yee, M.K., Travis, R.S., Goldhaber, S.Z., 2018c. Hypoalbuminemia and its association with venous thromboembolism in acutely ill hospitalized patients: Findings from the apex trial. Circulation 138.
31. Chi, G., Gibson, M., Hernandez, A.F., Hull, R.D., Cohen, A.T., Harrington, R.A., Liu, Y., Kalayci, A., Walia, S., Sharfaei, S., Pitliya, A., Kazmi, H.A., Datta, S., Kahe, F., Ghaffarpasand, E., Jafarizde, M., Yee, M.K., Travis, R.S., AlKhalfan, F., Nafee, T., Kerneis, M., Goldhaber, S.Z., 2018d. Prognostic value of C-reactive protein as inflammatory marker for venous thromboembolism in acutely ill hospitalized patients: Analysis from the apex trial. Circulation 138.
32. Chi, G., Gibson, C.M., Liu, Y., Hernandez, A.F., Hull, R.D., Cohen, A.T., Harrington, R.A., Goldhaber, S.Z., 2019. Inverse relationship of serum albumin to the risk of venous thromboembolism among acutely ill hospitalized patients: Analysis from the APEX trial. American Journal of Hematology 94, 21–28.
33. Chi, G., Violi, F., Pignatelli, P., Vestri, A., Spagnoli, A., Loffredo, L., Hernandez, A.F., Hull, R.D., Cohen, A.T., Harrington, R.A., Goldhaber, S.Z., Gibson, C.M., 2023. External validation of the ADA score for predicting thrombosis among acutely ill hospitalized medical patients from the APEX Trial. Journal of Thrombosis & Thrombolysis 55, 211–221.
34. Chulkov, V.S., Minina, E.E., Chulkov, V., Tkachenko, P.E., 2022. Obesity and risk of venous thromboembolic events. Profilakticheskaya Meditsina 25, 54–60. https://doi.org/10.17116/profmed20222510154
35. Cohen, A.T., Turpie, A.G.G., Leizorovicz, A., Olsson, C.-G., Vaitkus, P.T., Goldhaber, S.Z., Prevent Medical Thromboprophylaxis Study Group, 2007. Thromboprophylaxis with dalteparin in medical patients: which patients benefit? Vascular medicine (London, England) 12, 123–7. https://doi.org/10.1177/1358863X07079017
36. Cohen, A.T., Spiro, T.E., Buller, H.R., Haskell, L., Hu, D.Y., Hull, R., Mebazaa, A., Merli, G., Schellong, S., Spyropoulos, A., Tapson, V., 2010. Rivaroxaban compared with enoxaparin for the prevention of venous thromboembolism in acutely ill medical patients: MAGELLAN study methodology. Blood 116.
37. Cohen, A.T., Rietbrock, S., Martinez, C., 2011a. Risk factors for venous thromboembolism (VTE) in medical patients in the VTE epidemiology group (VEG) study. Journal of Thrombosis and Haemostasis 9, 750. https://doi.org/10.1111/j.1538-7836.2011.04380_4.x
38. Cohen, A.T., Spiro, T.E., Buller, H.R., Haskell, L., Hu, D.Y., Hull, R., Mebazaa, A., Merli, G., Schellong, S., Spyropoulos, A., Tapson, V., 2011b. Rivaroxaban vs. enoxaparin for the prevention of venous thromboembolism in acutely ill medical patients: Magellan subgroup analyses. Journal of Thrombosis and Haemostasis 9, 21.  https://doi.org/[10.1111/j.1538-7836.2011.04380_1.x](https://dx.doi.org/10.1111/j.1538-7836.2011.04380_1.x)
39. Cohen, A. T., Rietbrock, S., Martinez, C.2011c. Social risk factors for venous thromboembolism (VTE) in the VTE epidemiology group (VEG) study. Journal of Thrombosis and Haemostasis 9, 767. https://doi.org/[10.1111/j.1538-7836.2011.04380_4.x](https://dx.doi.org/10.1111/j.1538-7836.2011.04380_4.x)
40. Cohen, A.T., Spiro, T.E., Spyropoulos, A.C., Desanctis, Y.H., Homering, M., Buller, H.R., Haskell, L., Hu, D., Hull, R., Mebazaa, A., Merli, G., Schellong, S., Tapson, V.F., Burton, P., 2014. D-dimer as a predictor of venous thromboembolism in acutely ill, hospitalized patients: A subanalysis of the randomized controlled MAGELLAN trial. Journal of Thrombosis and Haemostasis 12, 479–487. https://doi.org/10.1111/jth.12515
41. Cohoon, K.P., De Sanctis, Y., Haskell, L., McBane, R.D., Spiro, T.E., 2018. Rivaroxaban for thromboprophylaxis among patients recently hospitalized for acute infectious diseases: a subgroup analysis of the MAGELLAN study. Journal of thrombosis and haemostasis : JTH 16, 1278–1287. https://doi.org/10.1111/jth.14146
42. Drozdinsky, G., Zusman, O., Kushnir, S., Leibovici, L., Gafter-Gvili, A., 2024. The effect of obligatory Padua prediction scoring in hospitalized medically ill patients: A retrospective cohort study. PLoS ONE 19, e0292661. https://doi.org/10.1371/journal.pone.0292661
43. Edelsberg, J., Hagiwara, M., Taneja, C., Oster, G., 2006. Risk of venous thromboembolism among hospitalized medically ill patients. American journal of health-system pharmacy : AJHP : official journal of the American Society of Health-System Pharmacists 63, S16-22. https://doi.org/10.2146/ajhp060389
44. Ferreira, C.R.L., de Bastos, M., Diniz, M.L., Mancini, R.A., Raposo, Y.S., Alves, S.M.P.G., Rezende, S.M., 2021. Inter-observer reliability of a risk assessment model for venous thromboembolism in acutely-ill medical hospitalized patients: Results from a prospective cohort study. Phlebology 36, 827–834. https://doi.org/10.1177/02683555211021226
45. Gal, G.L., Agnelli, G., Darius, H., Kahn, S.R., Owaidah, T., Rocha, A.T., Zhai, Z., Khan, I., Djoudi, Y., Ponomareva, E., Cohen, A.T., 2024. Event rates and risk factors for venous thromboembolism and major bleeding in a population of hospitalized adult patients with acute medical illness receiving enoxaparin thromboprophylaxis. European Journal of Internal Medicine 121, 48–55. https://doi.org/10.1016/j.ejim.2023.11.017
46. Gerotziafas, G.T., Chrysanthidis, M., Isaad, R., Baccouche, H., Papageorgiou, C., Thiolier, B., Spyropoulos, A.C., Galea, V., Katsamouris, A., Kiskinis, D., Elalamy, I., 2010. Prevalence of risk factors for VTE in hospitalized medical and surgical patients. Data from the comparison of methods for thromboembolic risk assessment with clinical perceptions and AwareneSS in real life surgical and medical patients (COMPASS) study. Blood 116.
47. Gibson, C., Chi, G., Jain, P., Szlosek, D., Goldhaber, S., Hull, R., Hernandez, A., Korjian, S., Daaboul, Y., Harrington, R., et al., 2016. Improvedd score: addition of d-dimer to the improve score improves venous thromboembolism risk stratification. an apex trial substudy. Circulation 134.
48. Grant, P., Greene, M.T., Bernstein, S.J., Wietzke, J.N., Cowan, K., McLaughlin, E., Kaatz, S., Paje, D., Lee, B., Barron, J., Flanders, S., 2013. Timing of post-discharge venous thromboembolic events and effect of prophylaxis in hospitalized medicine patients. Journal of General Internal Medicine 28, S222–S223.
49. Grant, P.J., Greene, M.T., Chopra, V., Bernstein, S.J., Hofer, T.P., Flanders, S.A., 2016. Assessing the Caprini Score for Risk Assessment of Venous Thromboembolism in Hospitalized Medical Patients. Am J Med 129, 528–535. https://doi.org/10.1016/j.amjmed.2015.10.027
50. Hotoleanu, C., Andercou, A., 2014. Risk factors in venous thromboembolism in hospitalized patients. Central European Journal of Medicine 9, 729–735. https://doi.org/10.2478/s11536-013-0324-9
51. Houghton, D.E., Desarno, M., Callas, P., Repp, A.B., Cushman, M., Merrill, S.A., Winters, I.J.P., Zakai, N.A., 2013. Validation of medical inpatient venous thrombosis risk assessment (MITH) score. Blood 122.
52. Hull, R., Bates, D., Brocklebank, C., Komari, N., Merali, T., 2010. Rate of late venous thromboembolism events in high-risk medical patients. Blood 116.
53. Kakkar, A., Cimminiello, C., Goldhaber, S., Parakh, R., Wang, C., Bergmann, J.-F., 2011. The impact of low-molecular-weight heparin prophylaxis on mortality in acutely ill medical patients: the LIFENOX study. Journal of thrombosis and haemostasis : JTH 9, 22. https://doi.org/10.1111/j.1538-7836.2011.04380_1.x
54. Kalayci, A., Gibson, C.M., Hernandez, A.F., Hull, R.D., Cohen, A.T., Fitzgerald, C., Hussain, S.D., Chi, G., Alkhalfan, F., Harrington, R.A., Goldhaber, S.Z., 2022. Inverse relationship between body mass index and risk of venous thromboembolism among medically ill hospitalized patients: Observations from the APEX trial. Thrombosis Research 211, 63–69. https://doi.org/10.1016/j.thromres.2022.01.016
55. Kato, S., Shimada, Y.J., Friedmann, P., Kashan, G., Husk, G., Bergmann, S.R., 2012. Identification of residual risk factors for the development of venous thromboembolism in medical inpatients receiving subcutaneous heparin therapy for prophylaxis. Coronary Artery Disease 23, 294–297. https://doi.org/10.1097/MCA.0b013e328352e510
56. Khorana, A.A., Francis, C.W., Culakova, E., Kuderer, N.M., Lyman, G.H., 2007. Frequency, risk factors, and trends for venous thromboembolism among hospitalized cancer patients. Cancer 110, 2339–46. https://doi.org/10.1002/cncr.23062
57. Kolomansky, A., Hoffman, R., Sarig, G., Brenner, B., Haim, N., 2006. Prospective evaluation of patients hospitalized with venous thromboembolism: comparison between cancer and non-cancer patients. The Israel Medical Association journal : IMAJ 8, 848–52.
58. Louzada, M.L., Kovacs, M.J., Al-Ani, F., Lazo-Langner, A., Siqueira, L., 2014. Assessment of risk of thromboprophylaxis failure in hospitalized patients with cancer (Artic Study). Blood 124.
59. Mahan, C.E., Vu, J., Spyropoulos, A.C., 2013. Validation study of the IMPACT-ILL venous thromboembolism risk assessment model in the acutely ill medical patient. Journal of Thrombosis and Haemostasis 11, 215–216.
60. Millar, J.A., Lett, J.E., Bagley, L.J., Densie, I.K., 2012. Eligibility for medical thromboprophylaxis based on risk-factor weights, and clinical thrombotic event rates. Medical Journal of Australia 196, 457–461.
61. Mottier, D., Girard, P., Couturaud, F., Lacut, K., Le Moigne, E., Paleiron, N., Guellec, D., Sanchez, O., Cogulet, V., Laporte, S., Marhic, G., Mismetti, P., Presles, E., Robert-Ebadi, H., Mahe, I., Plaisance, L., Reny, J.-L., Darbellay Farhoumand, P., Cuvelier, C., Le Henaff, C., Lambert, Y., Danguy des Deserts, M., Rousseau Legrand, C., Boutreux, S., Bleher, Y., Decours, R., Trinh-Duc, A., Armengol, G., Benhamou, Y., Daumas, A., Guyot, S.-L., De Carvalho, H., Lamia, B., Righini, M., Meyer, G., Le Gal, G., 2023. Enoxaparin versus Placebo to Prevent Symptomatic Venous Thromboembolism in Hospitalized Older Adult Medical Patients. NEJM evidence 2, EVIDoa2200332. https://doi.org/10.1056/EVIDoa2200332
62. Nafee, T., Gibson, C.M., Travis, R., Yee, M.K., Kerneis, M., Chi, G., AlKhalfan, F., Hernandez, A.F., Hull, R.D., Cohen, A.T., Harrington, R.A., Goldhaber, S.Z., 2020. Machine learning to predict venous thrombosis in acutely ill medical patients. Research and Practice in Thrombosis and Haemostasis 4, 230–237. https://doi.org/10.1002/rth2.12292
63. Neuman, R., Amin, A., Lingohr-Smith, M., Menges, B., Lin, J., 2017. The risk of venous thromboembolism and prophylaxis in the inpatient and outpatient continuum of care among acutely ill medical patients in the United States. Neurocritical Care 27, S235. https://doi.org/10.1007/s12028-017-0465-9
64. Novelli, E.M., Huynh, C., Gladwin, M.T., Moore, C.G., Ragni, M.V., 2012. Pulmonary embolism in sickle cell disease: a case-control study. Journal of thrombosis and haemostasis : JTH 10, 760–6. https://doi.org/10.1111/j.1538-7836.2012.04697.x
65. Perez, S.G., Ruiz-Talero, P., Velandia, O.M.M., 2023. Factors associated with venous thromboembolic disease due to failed thromboprophylaxis. Thrombosis Journal 21, 120. https://doi.org/10.1186/s12959-023-00566-4
66. Piazza, G., Goldhaber, S.Z., Lessard, D.M., Goldberg, R.J., Emery, C., Spencer, F.A., 2011. Venous thromboembolism in heart failure: preventable deaths during and after hospitalization. The American journal of medicine 124, 252–9. https://doi.org/10.1016/j.amjmed.2010.10.014
67. Prandoni, P., Barbar, S., Noventa, F., Rossetto, V., Ferrari, A., Brandolin, B., Perlati, M., De Bon, E., Tormene, D., Pagnan, A., Mannucci, P.M., 2010. A risk assessment model for the identification of hospitalized medical patients at risk for venous thromboembolism. Pathophysiology of Haemostasis and Thrombosis 37, A12. https://doi.org/10.1159/000318095
68. Rodrigues Lima Ferreira, C., Diniz, M.M., Avelino Mancini, R., Da Silva Raposo, Y., Miriam Paulinelli Garcia Alves, S., De Bastos, M., Meireles Rezende, S., 2019. Risk factors and incidence of venous thromboembolism in acutely-ill clinical hospitalized patients: Results of a prospective cohort study on thromboprophylaxis. Research and Practice in Thrombosis and Haemostasis 3, 890–891. https://doi.org/10.1002/rth2.12229
69. Rojnuckarin, P., Uaprasert, N., Vajragupta, L., Numkarunarunrote, N., Tanpowpong, N., Sutcharitchan, P., 2011. Risk factors for symptomatic venous thromboembolism in Thai hospitalised medical patients. Thrombosis and Haemostasis 106, 1103–8. https://doi.org/10.1160/TH11-08-0555
70. Rosenberg, D., Eichorn, A., Alarcon, M., McCullagh, L., McGinn, T., Spyropoulos, A.C., 2014. External validation of the risk assessment model of the International Medical Prevention Registry on Venous Thromboembolism (IMPROVE) for medical patients in a tertiary health system. Journal of the American Heart Association 3, e001152. https://doi.org/10.1161/JAHA.114.001152
71. Rothberg, M.B., Hamilton, A., Kou, L., Hu, B., Pappas, M.A., 2018. Development and validation of a risk assessment model for vte in hospitalized medical patients. Journal of General Internal Medicine 33, 156–157.
72. Rothberg, M.B., Hamilton, A.C., Greene, M.T., Fox, J., Lisheba, O., Milinovich, A., Gautier, T.N., Kim, P., Kaatz, S., Hu, B., 2022. Derivation and Validation of a Risk Factor Model to Identify Medical Inpatients at Risk for Venous Thromboembolism. Thrombosis and Haemostasis 122, 1231–1238. https://doi.org/10.1055/a-1698-6506
73. Ryan, L., Mataraso, S., Siefkas, A., Pellegrini, E., Barnes, G., Green-Saxena, A., Hoffman, J., Calvert, J., Das, R., 2021. A Machine Learning Approach to Predict Deep Venous Thrombosis Among Hospitalized Patients. Clinical and applied thrombosis/hemostasis : official journal of the International Academy of Clinical and Applied Thrombosis/Hemostasis 27, 1076029621991185. https://doi.org/10.1177/1076029621991185
74. Sejrup, J.K., Borvik, T., Grimnes, G., Isaksen, T., Hindberg, K., Hansen, J.-B., Morelli, V.M., Braekkan, S.K., 2019. Myocardial Infarction as a Transient Risk Factor for Incident Venous Thromboembolism: Results from a Population-Based Case-Crossover Study. Thrombosis and Haemostasis 119, 1358–1364. https://doi.org/10.1055/s-0039-1692176
75. Serrano, J., Rodriguez, B., Chindamo, M.C., 2018. IVSS safety zone protocol versus Padua score in the prevention of thromboembolic disease in medical patients: A comparative prospective study. Research and Practice in Thrombosis and Haemostasis 2, 326. https://doi.org/10.1002/rth2.12125
76. Shah, P., Arora, S., Kumar, V., Sharma, S., Shah, H., Tripathi, B., Sharma, P., Sharma, R., Savani, S., Qureshi, M.R., Faruqi, I., 2018. Short-term outcomes of pulmonary embolism: A National Perspective. Clinical cardiology 41, 1214–1224. https://doi.org/10.1002/clc.23048
77. Silvestri, F., Pasca, S., Zaramella, M., Labombarda, A., Barbi, A., Desideri, M., Guidi, P., Rogato, A., Bergamo, M., Mansutti, E., Barillari, G., 2013. Fondaparinux for the prevention of venous thromboembolism in elderly acutely ill medical patients with renal impairment: A retrospective single center cohort study. Italian Journal of Medicine 7, 113–114.
78. Smilowitz, N.R., Zhao, Q., Wang, L., Shrestha, S., Baser, O., Berger, J.S., 2019. Risk of Venous Thromboembolism after New Onset Heart Failure. Scientific reports 9, 17415. https://doi.org/10.1038/s41598-019-53641-0
79. Smilowitz, N.R., Subashchandran, V., Newman, J., Barfield, M.E., Maldonado, T.S., Brosnahan, S.B., Yuriditsky, E., Horowitz, J.M., Shah, B., Reynolds, H.R., Hochman, J.S., Berger, J.S., 2021. Risk of thrombotic events after respiratory infection requiring hospitalization. Scientific reports 11, 4053. https://doi.org/10.1038/s41598-021-83466-9
80. Sparks, A., Wilkinson, K., Repp, A., Li, A., Thomas, R., Roetker, N., Zakai, N., 2022. Validation of the IMPROVE hospital-acquired venous thrombosis risk assessment model in the medical inpatients thrombosis and hemostasis study (MITH) population. Research and Practice in Thrombosis and Haemostasis 6. https://doi.org/10.1002/rth2.12787
81. Spyropoulos, A.C., 2009. Venous thromboembolism risk factors in acutely ill hospitalized medical patients. Haematologica Meeting Reports 3, 21–22.
82. Spyropoulos, A.C., Anderson Jr, F.A., FitzGerald, G., Decousus, H., Pini, M., Chong, B.H., Zotz, R.B., Bergmann, J.F., Tapson, V., Froehlich, J.B., Monreal, M., Merli, G.J., Pavanello, R., Turpie, A.G.G., Nakamura, M., Piovella, F., Kakkar, A.K., Spencer, F.A., 2011. Predictive and associative models to identify hospitalized medical patients at risk for VTE. Chest 140, 706–714. https://doi.org/10.1378/chest.10-1944
83. Spyropoulos, A.C., Lipardi, C., Xu, J., Peluso, C., Spiro, T.E., De Sanctis, Y., Barnathan, E.S., Raskob, G.E., 2020. Modified IMPROVE VTE Risk Score and Elevated D-Dimer Identify a High Venous Thromboembolism Risk in Acutely Ill Medical Population for Extended Thromboprophylaxis. TH Open 4, E59–E65. https://doi.org/10.1055/s-0040-1705137
84. Sun, M.L., Wang, X.H., Huang, J., Wang, J., Wang, Y., 2018. [Comparative study on deep venous thrombosis onset in hospitalized patients with different underlying diseases]. Zhonghua nei ke za zhi 57, 429–434. https://doi.org/10.3760/cma.j.issn.0578-1426.2018.06.007
85. Thomas, R., Wilkinson, K., Sparks, A., Gergi, M., Repp, A., Roetker, N., Smith, N., Muthukrishnan, P., Zakai, N., 2023. Risk Factors for Venous Thrombosis after Discharge from Medical Hospitalizations: The Medical Inpatient Thrombosis and Hemostasis (MITH) Study. Research and Practice in Thrombosis and Haemostasis 7, 100644. https://doi.org/10.1016/j.rpth.2023.100644
86. Tsai, J., Grant, A.M., Beckman, M.G., Grosse, S.D., Yusuf, H.R., Richardson, L.C., 2015. Determinants of venous thromboembolism among hospitalizations of US adults: a multilevel analysis. PLoS ONE 10, e0123842. https://doi.org/10.1371/journal.pone.0123842
87. Vardi, M., Ghanem-Zoubi, N.O., Zidan, R., Yurin, V., Bitterman, H., 2013. Venous thromboembolism and the utility of the Padua Prediction Score in patients with sepsis admitted to internal medicine departments. Journal of Thrombosis and Haemostasis 11, 467–473. https://doi.org/10.1111/jth.12108
88. Vincentelli, G.M., Timpone, S., Murdolo, G., Fusco Moffa, I., L’angiocola, P.D., Borgognoni, F., Monti, M., 2018. A new risk assessment model for the stratification of the thromboembolism risk in medical patients: the TEVere Score. Minerva Medica 109, 436–442.
89. Wang, T.-F., Wong, C.A., Milligan, P.E., Thoelke, M.S., Woeltje, K.F., Gage, B.F., 2014. Risk factors for inpatient venous thromboembolism despite thromboprophylaxis. Thromb Res 133, 25–29. https://doi.org/10.1016/j.thromres.2013.09.011
90. Wilkinson, K.S., Sparks, A.D., Gergi, M., Repp, A.B., Al-Samkari, H., Thomas, R., Roetker, N.S., Zakai, N.A., 2024. Validation of the International Medical Prevention Registry on Venous Thromboembolism (IMPROVE) risk scores for venous thromboembolism and bleeding in an independent population. Research And Practice In Thrombosis And Haemostasis 8.
91. Woller, S.C., Stevens, S.M., Jones, J. p, Evans, R.S., Lloyd, J.F., Aston, V.T., Elliott, C.G., Mannucci, P.M., 2010. Establishing the optimal risk score to best promote venous thromboembolism prophylaxis among hospitalized medical patients (“Risk-e VTE”). Pathophysiology of Haemostasis and Thrombosis 37, A13. https://doi.org/10.1159/000318095
92. Woller, S., Stevens, S., Snow, G., Lloyd, J., Bledsoe, J., Fazili, M., Horne, B., 2020. DERIVATION AND VALIDATION OF THE HA-VTE AND HA-MB INTERMOUNTAIN RISK SCORES FROM UBIQUITOUS CLINICAL BIOMARKERS TO PREDICT 90-DAY HOSPITAL-ASSOCIATED VENOUS THROMBOEMBOLISM AND MAJOR BLEEDING AMONG MEDICAL PATIENTS. Chest 158, A2452–A2453. https://doi.org/10.1016/j.chest.2020.09.034
93. Wurtz, M., Grove, E.L., Corraini, P., Adelborg, K., Sundboll, J., Komjathine Szepligeti, S., Horvath-Puho, E., Sorensen, H.T., 2020. Comorbidity and risk of venous thromboembolism after hospitalization for first-time myocardial infarction: A population-based cohort study. Journal of thrombosis and haemostasis : JTH 18, 1974–1985. https://doi.org/10.1111/jth.14865
94. Yang, S., Zhang, Y., Jiao, X., Liu, J., Wang, W., Kuang, T., Gong, J., Li, J., Yang, Y., 2023. Padua prediction score may be inappropriate for VTE risk assessment in hospitalized patients with acute respiratory conditions: A Chinese single-center cohort study. International journal of cardiology. Heart & vasculature 49, 101301. https://doi.org/10.1016/j.ijcha.2023.101301
95. Zakai, N.A., Wright, J., Cushman, M., 2004. Risk factors for venous thrombosis in medical inpatients: Validation of a thrombosis risk score. Journal of Thrombosis and Haemostasis 2, 2156–2161. https://doi.org/10.1111/j.1538-7836.2004.00991.x
96. Zakai, N., Callas, P., Repp, A., Cushman, M., 2011a. Development and testing of a risk assessment model for venous thrombosis in medical inpatients: The Medical Inpatients and Thrombosis (MITH) study score. Blood 118.
97. Zakai, N., Callas, P., Repp, A., Cushman, M., 2011b. Incidence and rate of venous thrombosis in medical inpatients: The medical inpatients and thrombosis (MITH) study. Blood 118.
98. Zakai, N.A., Callas, P.W., Repp, A.B., Cushman, M., 2012. Myocardial infarction is not a risk factor for hospital-acquired venous thrombosis: The medical inpatients thrombosis (MITH) study. Circulation 125.
99. Zakai, N.A., Callas, P.W., Repp, A.B., Cushman, M., 2013. Venous thrombosis risk assessment in medical inpatients: The medical inpatients and thrombosis (MITH) study. Journal of Thrombosis and Haemostasis 11, 634–641. https://doi.org/10.1111/jth.12147
100. Zakai, N.A., Koh, I., Wilkinson, K., Roetker, N.S., Sparks, A.D., Repp, A.B., Thomas, R.M., Smith, N.L., Holmes, C.E., Cushman, M., 2021. Risk assessing medical inpatients for hospital-acquired venous thrombosis: The medical inpatients thrombosis and hemostasis (MITH) study. Blood 138, 829. https://doi.org/10.1182/blood-2021-152584
101. Zakai, N.A., Wilkinson, K., Sparks, A.D., Packer, R.T., Koh, I., Roetker, N.S., Repp, A.B., Thomas, R., Holmes, C.E., Cushman, M., Plante, T.B., Al-Samkari, H., Pishko, A.M., Wood, W.A., Masias, C., Gangaraju, R., Li, A., Garcia, D., Wiggins, K.L., Schaefer, J.K., Hooper, C., Smith, N.L., McClure, L.A., 2024. Development and validation of a risk model for hospital-acquired venous thrombosis: the Medical Inpatients Thrombosis and Hemostasis study. Journal of thrombosis and haemostasis : JTH 22, 503–515. https://doi.org/10.1016/j.jtha.2023.10.015

Supplemental Table 3. Other studies excluded with rationale

|  | **Author, year** | **Reason for exclusion** |
| --- | --- | --- |
|  | Abenante et al., 2025 | Population: Not only hospitalised medical patients |
|  | Andishmand et al., 2024 | No analysis of risk factors associated with VTE |
|  | Angelini et al., 2015 | No analysis of risk factors associated with VTE |
|  | Bansal et al., 2025 | No analysis of risk factors associated with VTE |
|  | Brenner et al., 2024 | No analysis of risk factors associated with VTE |
|  | Calé et al., 2025 | No analysis of risk factors associated with VTE |
|  | Cao et al., 2024 | No analysis of risk factors associated with VTE |
|  | Chen et al., 2025 | Population: Not only hospitalised medical patients |
|  | Dh te et al., 2001 | Systematic review/review article |
|  | Farogh et al., 2024 | No analysis of risk factors associated with VTE |
|  | Gabet et al., 2024 | Systematic review/review article |
|  | Garg et al., 2025 | No analysis of risk factors associated with VTE |
|  | Keller et al., 2024 | Population: Not only hospitalised medical patients |
|  | Khan et al., 2024 | Population: Not only hospitalised medical patients |
|  | Kolkailah et al., 2024 | No analysis of risk factors associated with VTE |
|  | Kwok et al., 2025 | No analysis of risk factors associated with VTE |
|  | Lebow et al., 2015 | No analysis of risk factors associated with VTE |
|  | Lee and Er, 2025 | No analysis of risk factors associated with VTE |
|  | Li et al., 2024 | No analysis of risk factors associated with VTE |
|  | Li et al., 2025 | No analysis of risk factors associated with VTE |
|  | Mittman et al., 2024 | Population: Not only hospitalised medical patients |
|  | Neeman et al., 2022 | No analysis of risk factors associated with VTE |
|  | Raya-Benítez et al., 2025 | No analysis of risk factors associated with VTE |
|  | Repp et al., 2024 | No analysis of risk factors associated with VTE |
|  | Richardson et al., 2025 | Population: Not only hospitalised medical patients |
|  | Rincón Díaz et al., 2024 | No analysis of risk factors associated with VTE |
|  | Ruiz-Artacho et al., 2025 | No analysis of risk factors associated with VTE |
|  | Shapiro et al., 2025 | Population: Not only hospitalised medical patients |
|  | Sharma et al., 2025 | No analysis of risk factors associated with VTE |
|  | Singh et al., 2024 | Systematic review/review article |
|  | Sisi et al., 2024 | No analysis of risk factors associated with VTE |
|  | Thomas et al., 2025 | Systematic review/review article |
|  | Tsaftaridis et al., 2025 | No analysis of risk factors associated with VTE |
|  | Vladic et al., 2025 | No analysis of risk factors associated with VTE |
|  | Wang et al., 2024 | No analysis of risk factors associated with VTE |
|  | Wang et al., 2025 | No analysis of risk factors associated with VTE |
|  | Wolf et al., 2025 | No analysis of risk factors associated with VTE |
|  | Woller et al., 2024 | Population: Not only hospitalised medical patients |
|  | Ye et al., 2017 | Population: Not only hospitalised medical patients |
|  | Zaihan et al., 2025 | Population: Not only hospitalised medical patients |
|  | Zeng et al., 2025 | Population: Not only hospitalised medical patients |
|  | Zhang et al., 2025 | No analysis of risk factors associated with VTE |

- 1. Abenante, A., Squizzato, A., Bertù, L., Arioli, D., Buso, R., Carrara, D., Ciarambino, T., Dentali, F., 2025. Predictors for the prescription of pharmacological prophylaxis for venous thromboembolism during hospitalization in Internal Medicine: a sub-analysis of the FADOI-NoTEVole study. Intern Emerg Med 20, 151–158. https://doi.org/10.1007/s11739-024-03770-w
  2. Andishmand, A., Sharifi, L., Namayandeh, S.M., 2024. Clinical Profile and Outcomes of Pulmonary Embolism in Central Iran: A Retrospective Cohort Study. Arch Iran Med 27, 667–673. https://doi.org/10.34172/aim.31907
  3. Angelini, D.E., Greene, T., Wietzke, J.N., Flanders, S.A., Sood, S.L., 2015. Defining the Risk: Benefit Ratio of Venous Thromboembolism (VTE) Prophylaxis in Hospitalized Cancer Patients. Blood 126, 627. https://doi.org/10.1182/blood.V126.23.627.627
  4. Bansal, M., Mehta, A., Ahmad, K., Bortnick, A.E., Nagaraja, V., Hyder, O.N., Dawn Abbott, J., Vallabhajosyula, S., 2025. Management and Outcomes of Pulmonary Embolism in Women of Reproductive Age Admitted to Urban Versus Rural Areas Compared to Men. Catheter Cardiovasc Interv 105, 193–199. https://doi.org/10.1002/ccd.31344
  5. Brenner, B., Tzoran, I., Bikdeli, B., Valle, R., Poenou, G., Tirado-Miranda, R., Pesce, M.L., Pagán-Escribano, J., Giorgi-Pierfranceschi, M., Monreal, M., RIETE Investigators, Adarraga, M.D., Aibar, J., Alberich-Conesa, A., Alonso-Carrillo, J., Alda-Lozano, A., Alfonso, J., Amado, C., Angelina-García, M., Arcelus, J.I., Ballaz, A., Barba, R., Barbagelata, C., Barreiro, B., Barrón, M., Barrón-Andrés, B., Beddar-Chaib, F., Blanco-Molina, A., Caballero, J.C., Cantarella-Bongiovanni, R.F., Cañas, I., Carrero-Arribas, R., Casado, I., Castellanos, G., Criado, J., De Juana-Izquierdo, C., Del Toro, J., Demelo-Rodríguez, P., DÍaz-Brasero, A.M., DÍaz-Pedroche, M.C., DÍaz-Peromingo, J.A., Dubois-Silva, A., Escribano, J.C., Falgá, C., Fernández-Aracil, C., Fernández-Capitán, C., Fernández-Jiménez, B., Fernández-Reyes, J.L., Fidalgo, M.A., Francisco, I., 2024. Venous Thromboembolism after Hospital Discharge: Temporal Trends in Baseline Characteristics, Prevention, Treatment, and 90-day Outcomes. Seminars in Thrombosis and Hemostasis. https://doi.org/10.1055/s-0044-1791817
  6. Calé, R., Ascenção ,R., Bulhosa ,C., Pereira ,H., Borges ,M., Costa ,J., and Caldeira, D., 2025. In-hospital mortality of high-risk pulmonary embolism: a nationwide population-based cohort study in Portugal from 2010 to 2018. Pulmonology 31, 2416830. https://doi.org/10.1016/j.pulmoe.2023.11.002
  7. Cao, Z., Yang, L., Han, J., Lv, X., Wang, X., Zhang, B., Ye, X., Ye, H., 2024. Development of a predictive nomogram for early identification of pulmonary embolism in hospitalized patients: a retrospective cohort study. BMC Pulm Med 24, 594. https://doi.org/10.1186/s12890-024-03377-z
  8. Chen, B., Wang, W., Zang, W., Hu, J., Li, H., Wang, C., Zhu, Y., Mao, Y., Gao, P., 2025. Analysis of clinical characteristics in 1940 patients with acute pulmonary embolism. Respiratory Medicine 237, 107920. https://doi.org/10.1016/j.rmed.2024.107920
  9. Dh te, R., Pellicer-Coeuret, M., Belouet-Moreau, C., Christoforov, B., Vidal-Trecan, G., 2001. Venous thromboembolism in medical inpatients: prophylaxis with low-weight heparin in a university hospital and prevalence of thromboembolic events. Clin Appl Thromb Hemost 7, 16–20. https://doi.org/10.1177/107602960100700104
  10. Farogh, A., Wazir, N.U., Mumtaz, S., Salman, F., Arif, A., Umair, A., 2024. VENOUS THROMBOEMBOLISM RISK ASSESSMENT IN HOSPITALISED PATIENTS IN A TERTIARY CARE HOSPITAL IN PAKISTAN. J Ayub Med Coll Abbottabad 36, 383–387. https://doi.org/10.55519/JAMC-02-13243
  11. Gabet, A., Blacher, J., Tuppin, P., Lailler, G., Grave, C., Sanchez, O., Mahe, I., Emmerich, J., Olié, V., 2024. Epidemiology of venous thromboembolism in France. Archives of Cardiovascular Diseases, Update on epidemiology of cardiovascular risk factors and diseases in France 117, 715–724. https://doi.org/10.1016/j.acvd.2024.10.325
  12. Garg, A., Saleemi, A., Asfaw, M., Aldaoud, N., Chalasani, P., Lavu, V.K., Bhui, P., Nagar, T., Agarwal, A., Yesilyaprak, A., Kumar, J., Mansour, M., Bock, D., Nyongbella, J., Kulairi, Z., 2025. The outcomes of the obesity paradox in pulmonary embolism: a study of the national inpatient sample database from 2016 to 2020. Ann Hematol 104, 1187–1193. https://doi.org/10.1007/s00277-025-06197-1
  13. Keller, K., Sivanathan, V., Farmakis, I.T., Schmitt, V.H., Espinola-Klein, C., Schmidt, F.P., Münzel, T., Konstantinides, S., Hobohm, L., 2024. Incidence and impact of venous thromboembolism in hospitalized patients with acute pancreatitis. Dig Liver Dis 56, 2085–2094. https://doi.org/10.1016/j.dld.2024.06.023
  14. Khan, M.I., O’Brien, A., O’Leary, C., Silvari, V., Duggan, C., O’Shea, S., 2024. IMPROVING VTE RISK ASSESSMENT IN HOSPITALISED PATIENTS IN A TERTIARY CARE HOSPITAL IN IRELAND. J Ayub Med Coll Abbottabad 36, 125–130. https://doi.org/10.55519/JAMC-01-13044
  15. Kolkailah, A.A., Abdelghaffar, B., Elshafeey, F., Magdy, R., Kamel, M., Abuelnaga, Y., Nabhan^a^, A.F., Piazza^a^, G., 2024. Standard‐ versus extended‐duration anticoagulation for primary venous thromboembolism prophylaxis in acutely ill medical patients - Kolkailah, AA - 2024 | Cochrane Library.
  16. Kwok, C.S., Qureshi, A.I., Lin, Y., Liu, F., Holroyd, E., Lip, G.Y.H., Bradaric, A., Borovac, J.A., 2025. Incidence, predictors and outcomes associated with acute pulmonary embolism in patients hospitalized with pneumonia: Insights from the National Inpatient Sample 23, 181–190.
  17. Lebow, M.E., DeSarno, M., Houghton, D.E., Winters, J.P., Merrill, S.A., Atweh, G.F., Zakai, N.A., 2015. Relative and Absolute Platelet Count Drops As a Risk Factor for Mortality, Bleeding, and Venous Thrombosis in Hospitalized Medical Patients. Blood 126, 2258. https://doi.org/10.1182/blood.V126.23.2258.2258
  18. Lee, S.Y., Er, C., 2025. Venous Thromboembolism Risk Assessment and Thromboprophylaxis Practice in Hospitalized Medical Patients: The Experience of a Singapore Teaching Hospital. Int J Angiol 34, 75–77. https://doi.org/10.1055/s-0044-1791506
  19. Li, H.-L., Zhang, H., Chan, Y.C., Cheng, S.W., 2025. Prevalence and risk factors of hospital acquired venous thromboembolism. Phlebology 40, 266–274. https://doi.org/10.1177/02683555241297566
  20. Li, R., Zeng, J., Sun, D., Li, D., 2024. The challenges of identifying pulmonary embolism in patients hospitalized for exacerbations of COPD. Respiratory Medicine and Research 86, 101122. https://doi.org/10.1016/j.resmer.2024.101122
  21. Mittman, B.G., Hu, B., Schulte, R., Le, P., Pappas, M.A., Hamilton, A., Rothberg, M.B., 2024. What Constitutes High Risk for Venous Thromboembolism? Comparing Approaches to Determining an Appropriate Threshold. https://doi.org/10.1101/2024.08.30.24312871
  22. Neeman, E., Liu, V., Mishra, P., Thai, K.K., Xu, J., Clancy, H.A., Schlessinger, D., Liu, R., 2022. Trends and Risk Factors for Venous Thromboembolism Among Hospitalized Medical Patients. JAMA Netw Open 5, e2240373. https://doi.org/10.1001/jamanetworkopen.2022.40373
  23. Raya-Benítez, J., Heredia-Ciuró, A., Calvache-Mateo, A., Martín-Núñez, J., Valenza-Peña, G., López-López, L., Valenza, M.C., 2025. Effectiveness of non-instrumental early mobilization to reduce the incidence of deep vein thrombosis in hospitalized patients: A systematic review and meta-analysis. International Journal of Nursing Studies 161, 104917. https://doi.org/10.1016/j.ijnurstu.2024.104917
  24. Repp, A.B., Sparks, A.D., Wilkinson, K., Roetker, N.S., Schaefer, J.K., Li, A., McClure, L.A., Terrell, D.R., Ferraris, A., Adamski, A., Smith, N.L., Zakai, N.A., 2024. Factors associated with venous thromboembolism pharmacoprophylaxis initiation in hospitalized medical patients: the Medical Inpatients Thrombosis and Hemostasis study. Journal of Thrombosis and Haemostasis 22, 3521–3531. https://doi.org/10.1016/j.jtha.2024.08.016
  25. Richardson, J.S., Clark, C.L., Bastani, A., Shams, A.H., Fermann, G.J., Hiestand, B.C., Kea, B., Mace, S.E., Peacock, W.F., Yang, A., Welker, J.A., 2025. D-dimer Levels in Acute, Medically Ill, Hospitalized Patients: A Large, Prospective, Multicenter Study in the United States. Clin Appl Thromb Hemost 31, 10760296251320406. https://doi.org/10.1177/10760296251320406
  26. Rincón Díaz, C., Jiménez Hernández, S., Beddar Chaib, F., Condon Abanto, A.I., Cortés Ayaso, M., Lozano Polo, L., Guirado Torrecillas, L., Alonso Valle, H., Olid Velilla, M., Jiménez, D., Lecumberri, R., Ruiz Artacho, P., Grupo de trabajo de Enfermedad Tromboembólica Venosa de la Sociedad Española de Medicina de Urgencias y Emergencias (ETV-SEMES)*, 2024. Prophylaxis of venous thromboembolism in hospitalized patients admitted from Spanish emergency departments: the PROTESU II study. Emergencias 36, 271–280. https://doi.org/10.55633/s3me/051.2024
  27. Ruiz-Artacho, P., Olid Velilla, M., Beddar Chaib, F., Lecumberri, R., Jiménez, D., Hernández Castells, L., Alonso Valle, H., Pedraza García, J., Sendín Martín, V., Cárdenas Bravo, L., Muriel, A., Jiménez Hernández, S., ETV-SEMES Investigators, 2025. Comparative Validation of Risk Assessment Models for Venous Thromboembolism Risk in Hospitalized Medical Patients: Insights from a Multicenter Prospective Cohort Study. Am J Med S0002-9343(25)00204–9. https://doi.org/10.1016/j.amjmed.2025.03.027
  28. Shapiro, S., Majert, J., Obeidalla, A., Clift, A., Havord, S., Jebamani, A., Matejtschuk, C., Clarke, P., Lasserson, D., 2025. Same-day emergency care: a retrospective observational study of the incidence and predictors of venous thromboembolism following hospital-based acute ambulatory medical care. J Thromb Haemost 23, 97–107. https://doi.org/10.1016/j.jtha.2024.09.017
  29. Sharma, A., Isabelle, M., Hunsaker, A., Dutta, S., Lucier, D., Rosovsky, R.P., Saini, S., Landman, A., Raja, A.S., Khorasani, R., Lacson, R., 2025. Impact of a Clinical Decision Support System on CT Pulmonary Angiography Yield and Utilization in Hospitalized Patients. Journal of the American College of Radiology 22, 449–460. https://doi.org/10.1016/j.jacr.2024.11.030
  30. Singh, R.R., Thandassery, R.B., Chawla, S., 2024. Acute Venous Thromboembolism Is Common Following Acute Necrotizing Pancreatitis and Is Associated With Worse Clinical Outcomes. Pancreas 53, e802–e807. https://doi.org/10.1097/MPA.0000000000002375
  31. Sisi, Y., Genpeng, L., Yao, C., Suting, S., Rongying, T., Jiayi, D., Zhaoli, Z., Chunyu, W., 2024. A Nomogram for Predicting Cancer-Associated Venous Thromboembolism in Hospitalized Patients Receiving Chemoradiotherapy for Cancer. Cancer Control 31, 10732748241290767. https://doi.org/10.1177/10732748241290767
  32. Thomas, R.M., Sparks, A.D., Wilkinson, K., Gergi, M., Repp, A.B., Roetker, N.S., Smith, N.L., Muthukrishnan, P., Martin, K., Zakai, N.A., 2025. Risk factors for venous thrombosis after discharge from medical hospitalizations: the Medical Inpatient Thrombosis and Hemostasis study. J Thromb Haemost S1538-7836(25)00254–5. https://doi.org/10.1016/j.jtha.2025.04.005
  33. Tsaftaridis, N., Cholagh, A., Kaatz, S., Spyropoulos, A.C., 2025. Venous Thromboembolism Prevention in the Hospitalized Medical Patient. Medical Clinics 0. https://doi.org/10.1016/j.mcna.2025.02.003
  34. Vladic, N., Englisch, C., Ay, C., Pabinger, I., 2025. Risk assessment and prevention of cancer-associated venous thromboembolism in ambulatory patients with solid malignancies. Research and Practice in Thrombosis and Haemostasis 9, 102664. https://doi.org/10.1016/j.rpth.2024.102664
  35. Wang, X., Wang, J., Zhang, Xiaoming, Zhang, Xuemin, Li, Q., Li, W., Jiang, J., Jiao, Y., Zhang, T., 2025. Trends of Hospital-Acquired Lower Extremity Deep Venous Thrombosis in an Academic Medical Center in China from 2007 to 2021. Annals of Vascular Surgery 115, 3–12. https://doi.org/10.1016/j.avsg.2024.12.072
  36. Wang, X., Yang, Y.-Q., Hong, X.-Y., Liu, S.-H., Li, J.-C., Chen, T., Shi, J.-H., 2024. A new risk assessment model of venous thromboembolism by considering fuzzy population. BMC Medical Informatics and Decision Making 24, 413. https://doi.org/10.1186/s12911-024-02834-3
  37. Wolf, S., Valerio, L., Fumagalli, R.M., Konstantinides, S.V., Ulrich, S., Klok, F.A., Cannegieter, S.C., Kucher, N., Barco, S., 2025. Acute pulmonary embolism with and without hemodynamic instability (2003-2022): a Swiss nationwide epidemiologic study. Journal of Thrombosis and Haemostasis 23, 1340–1351. https://doi.org/10.1016/j.jtha.2024.12.040
  38. Woller, S.C., Stevens, S.M., Bledsoe, J.R., Hellewell, J., Kraft, A., Butler, A.M., Fazili, M., Lloyd, J.F., Christensen, P.S., Peltan, I.D., Barnes, G.D., Horne, B.D., 2024. Methods of a cluster-randomized, type II hybrid implementation effectiveness trial to prospectively assess extended-duration thromboprophylaxis for at-risk medical patients being discharged to prevent hospital-associated venous thromboembolism. Res Pract Thromb Haemost 8, 102549. https://doi.org/10.1016/j.rpth.2024.102549
  39. Ye, F., Stalvey, C., Khuddus, M.A., Winchester, D.E., Toklu, H.Z., Mazza, J.J., Yale, S.H., 2017. A systematic review of mobility/immobility in thromboembolism risk assessment models for hospitalized patients. J Thromb Thrombolysis 44, 94–103. https://doi.org/10.1007/s11239-017-1501-5
  40. Zaihan, A.F., Wong, J.N., Kow, C.S., 2025. Pharmacological Venous Thromboembolism Prophylaxis Practices in Hospitalized Elderly Patients: a Retrospective Study in Malaysia. SN Compr. Clin. Med. 7, 46. https://doi.org/10.1007/s42399-025-01802-w
  41. Zeng, J., Feng, J., Luo, Y., Wei, H., Ge, H., Liu, H., Zhang, Jianchu, Li, X., Pan, P., Xie, X., Yi, M., Cheng, L., Zhou, Hui, Zhang, Jiarui, Peng, L., Pu, J., Chen, X., Yi, Q., Zhou, Haixia, MAGNET AECOPD Registry Investigators, 2025. Inflammatory Biomarkers as Predictors of Symptomatic Venous Thromboembolism in Hospitalized Patients with AECOPD: A Multicenter Cohort Study. J Atheroscler Thromb 32, 439–457. https://doi.org/10.5551/jat.65177
  42. Zhang, R.S., Yuriditsky, E., Zhang, P., Taslakian, B., Elbaum, L., Greco, A.A., Mukherjee, V., Postelnicu, R., Amoroso, N.E., Maldonado, T.S., Horowitz, J.M., Bangalore, S., 2025. Impact of Time to Catheter-Based Therapy on Outcomes in Acute Pulmonary Embolism. Circ Cardiovasc Interv 18, e014499. https://doi.org/10.1161/CIRCINTERVENTIONS.124.014499

Supplementary Table 4: PRISMA Checklist 2020

| **Section and Topic** | **Item #** | **Checklist item** | **Location where item is reported** |
| --- | --- | --- | --- |
| **TITLE** | | |  |
| Title | 1 | Identify the report as a systematic review. | P1 |
| **ABSTRACT** | | |  |
| Abstract | 2 | See the PRISMA 2020 for Abstracts checklist. | P3 |
| **INTRODUCTION** | | |  |
| Rationale | 3 | Describe the rationale for the review in the context of existing knowledge. | P4 |
| Objectives | 4 | Provide an explicit statement of the objective(s) or question(s) the review addresses. | P4 |
| **METHODS** | | |  |
| Eligibility criteria | 5 | Specify the inclusion and exclusion criteria for the review and how studies were grouped for the syntheses. | P5-6 |
| Information sources | 6 | Specify all databases, registers, websites, organisations, reference lists and other sources searched or consulted to identify studies. Specify the date when each source was last searched or consulted. | P5, supplementary info |
| Search strategy | 7 | Present the full search strategies for all databases, registers and websites, including any filters and limits used. | Supplementary info |
| Selection process | 8 | Specify the methods used to decide whether a study met the inclusion criteria of the review, including how many reviewers screened each record and each report retrieved, whether they worked independently, and if applicable, details of automation tools used in the process. | P3 |
| Data collection process | 9 | Specify the methods used to collect data from reports, including how many reviewers collected data from each report, whether they worked independently, any processes for obtaining or confirming data from study investigators, and if applicable, details of automation tools used in the process. | P5 |
| Data items | 10a | List and define all outcomes for which data were sought. Specify whether all results that were compatible with each outcome domain in each study were sought (e.g. for all measures, time points, analyses), and if not, the methods used to decide which results to collect. | P7 |
|  | 10b | List and define all other variables for which data were sought (e.g. participant and intervention characteristics, funding sources). Describe any assumptions made about any missing or unclear information. | P7 |
| Study risk of bias assessment | 11 | Specify the methods used to assess risk of bias in the included studies, including details of the tool(s) used, how many reviewers assessed each study and whether they worked independently, and if applicable, details of automation tools used in the process. | P7 |
| Effect measures | 12 | Specify for each outcome the effect measure(s) (e.g. risk ratio, mean difference) used in the synthesis or presentation of results. | P7 |
| Synthesis methods | 13a | Describe the processes used to decide which studies were eligible for each synthesis (e.g. tabulating the study intervention characteristics and comparing against the planned groups for each synthesis (item #5)). | P7 |
|  | 13b | Describe any methods required to prepare the data for presentation or synthesis, such as handling of missing summary statistics, or data conversions. | P7 |
|  | 13c | Describe any methods used to tabulate or visually display results of individual studies and syntheses. | P7 |
|  | 13d | Describe any methods used to synthesize results and provide a rationale for the choice(s). If meta-analysis was performed, describe the model(s), method(s) to identify the presence and extent of statistical heterogeneity, and software package(s) used. | P7 |
|  | 13e | Describe any methods used to explore possible causes of heterogeneity among study results (e.g. subgroup analysis, meta-regression). | P7 |
|  | 13f | Describe any sensitivity analyses conducted to assess robustness of the synthesized results. | P7 |
| Reporting bias assessment | 14 | Describe any methods used to assess risk of bias due to missing results in a synthesis (arising from reporting biases). | P7 |
| Certainty assessment | 15 | Describe any methods used to assess certainty (or confidence) in the body of evidence for an outcome. | P7 |
| **RESULTS** | | |  |
| Study selection | 16a | Describe the results of the search and selection process, from the number of records identified in the search to the number of studies included in the review, ideally using a flow diagram. | P8 |
|  | 16b | Cite studies that might appear to meet the inclusion criteria, but which were excluded, and explain why they were excluded. | P8 |
| Study characteristics | 17 | Cite each included study and present its characteristics. | P8-16 |
| Risk of bias in studies | 18 | Present assessments of risk of bias for each included study. | P17-18 |
| Results of individual studies | 19 | For all outcomes, present, for each study: (a) summary statistics for each group (where appropriate) and (b) an effect estimate and its precision (e.g. confidence/credible interval), ideally using structured tables or plots. | P18-19 |
| Results of syntheses | 20a | For each synthesis, briefly summarise the characteristics and risk of bias among contributing studies. | P18 |
|  | 20b | Present results of all statistical syntheses conducted. If meta-analysis was done, present for each the summary estimate and its precision (e.g. confidence/credible interval) and measures of statistical heterogeneity. If comparing groups, describe the direction of the effect. | P18-22 |
|  | 20c | Present results of all investigations of possible causes of heterogeneity among study results. | P23 |
|  | 20d | Present results of all sensitivity analyses conducted to assess the robustness of the synthesized results. | P8-9 |
| Reporting biases | 21 | Present assessments of risk of bias due to missing results (arising from reporting biases) for each synthesis assessed. | P17-18 |
| Certainty of evidence | 22 | Present assessments of certainty (or confidence) in the body of evidence for each outcome assessed. | P23 |
| **DISCUSSION** | | |  |
| Discussion | 23a | Provide a general interpretation of the results in the context of other evidence. | P24-25 |
|  | 23b | Discuss any limitations of the evidence included in the review. | P25 |
|  | 23c | Discuss any limitations of the review processes used. | P25 |
|  | 23d | Discuss implications of the results for practice, policy, and future research. | P25-26 |
| **OTHER INFORMATION** | | |  |
| Registration and protocol | 24a | Provide registration information for the review, including register name and registration number, or state that the review was not registered. | P3 |
|  | 24b | Indicate where the review protocol can be accessed, or state that a protocol was not prepared. | P3 |
|  | 24c | Describe and explain any amendments to information provided at registration or in the protocol. | - |
| Support | 25 | Describe sources of financial or non-financial support for the review, and the role of the funders or sponsors in the review. | P2 |
| Competing interests | 26 | Declare any competing interests of review authors. | P2 |
| Availability of data, code and other materials | 27 | Report which of the following are publicly available and where they can be found: template data collection forms; data extracted from included studies; data used for all analyses; analytic code; any other materials used in the review. | - |
